# Identifying pathways modulating sleep duration: from genomics to transcriptomics

**DOI:** 10.1038/s41598-017-04027-7

**Published:** 2017-07-04

**Authors:** Karla V. Allebrandt, Maris Teder-Laving, Paola Cusumano, Goar Frishman, Rosa Levandovski, Andreas Ruepp, Maria P. L. Hidalgo, Rodolfo Costa, Andres Metspalu, Till Roenneberg, Cristiano De Pittà

**Affiliations:** 1 Institute of Medical Psychology, Ludwig-Maximilians-University, Munich, Germany; 2 Estonian Genome Center and Institute of Molecular and Cell Biology of University of Tartu, Estonian Biocentre, Tartu, Estonia; 3 Department of Biology, University of Padova, Padova, Italy; 4 Institute for Bioinformatics and Systems Biology (IBIS); Helmholtz Center Munich, German Research Center for Environmental Health (GmbH), Neuherberg, Germany; 5 Departamento de Psiquiatria e Medicina Legal, Universidade Federal do Rio Grande do Sul (UFRGS), Porto Alegre, Brazil

## Abstract

Recognizing that insights into the modulation of sleep duration can emerge by exploring the functional relationships among genes, we used this strategy to explore the genome-wide association results for this trait. We detected two major signalling pathways (ion channels and the ERBB signalling family of tyrosine kinases) that could be replicated across independent GWA studies meta-analyses. To investigate the significance of these pathways for sleep modulation, we performed transcriptome analyses of short sleeping flies’ heads (knockdown for the *ABCC9* gene homolog; d*Sur*). We found significant alterations in gene-expression in the short sleeping knockdowns *versus* controls flies, which correspond to pathways associated with sleep duration in our human studies. Most notably, the expression of *Rho* and *EGFR* (members of the ERBB signalling pathway) genes was down- and up-regulated, respectively, consistently with the established role of these genes for sleep consolidation in *Drosophila*. Using a disease multifactorial interaction network, we showed that many of the genes of the pathways indicated to be relevant for sleep duration had functional evidence of their involvement with sleep regulation, circadian rhythms, insulin secretion, gluconeogenesis and lipogenesis.

## Introduction

Environmental cues, such as time of year and amplitude of seasonal changes, influence sleep behaviour^1^, neurobehavioral disorders^2^ and energy metabolism, supporting the link between these systems. Too little or too much sleep is also associated with health deficits, including high body-mass index (BMI), hypertension, cardiovascular diseases, type–2 diabetes, and general mortality^3^. Although the underlying mechanisms are not yet known, homeostatic and circadian sleep regulation interactively influence energy metabolism^4^ and mood^2^.

In our earlier reported meta-analysis for genome-wide association studies (GWAS; N = 4251) on sleep duration, we identified one genome-wide significant signal (rs11046205) in the *ABCC9* (ATP-binding cassette, sub-family C, member 9) gene^5^. *ABCC9* encodes a pore-forming subunit (SUR2) of an ATP-sensitive potassium channel (K_ATP_), a sulfonylurea receptor which plays a role in the aetiology of cardiomyopathies and energy metabolism^6^. By knocking down its homologue (d*Sur*, Sulfonylurea receptor) in *Drosophila*, we earlier reported a shortening in night sleep in this organism^5^. *ABCC9* gene expression alterations in a sub-population of neurons activated during sleep deprivation in mice were previously reported^7^.

**Figure 1:**
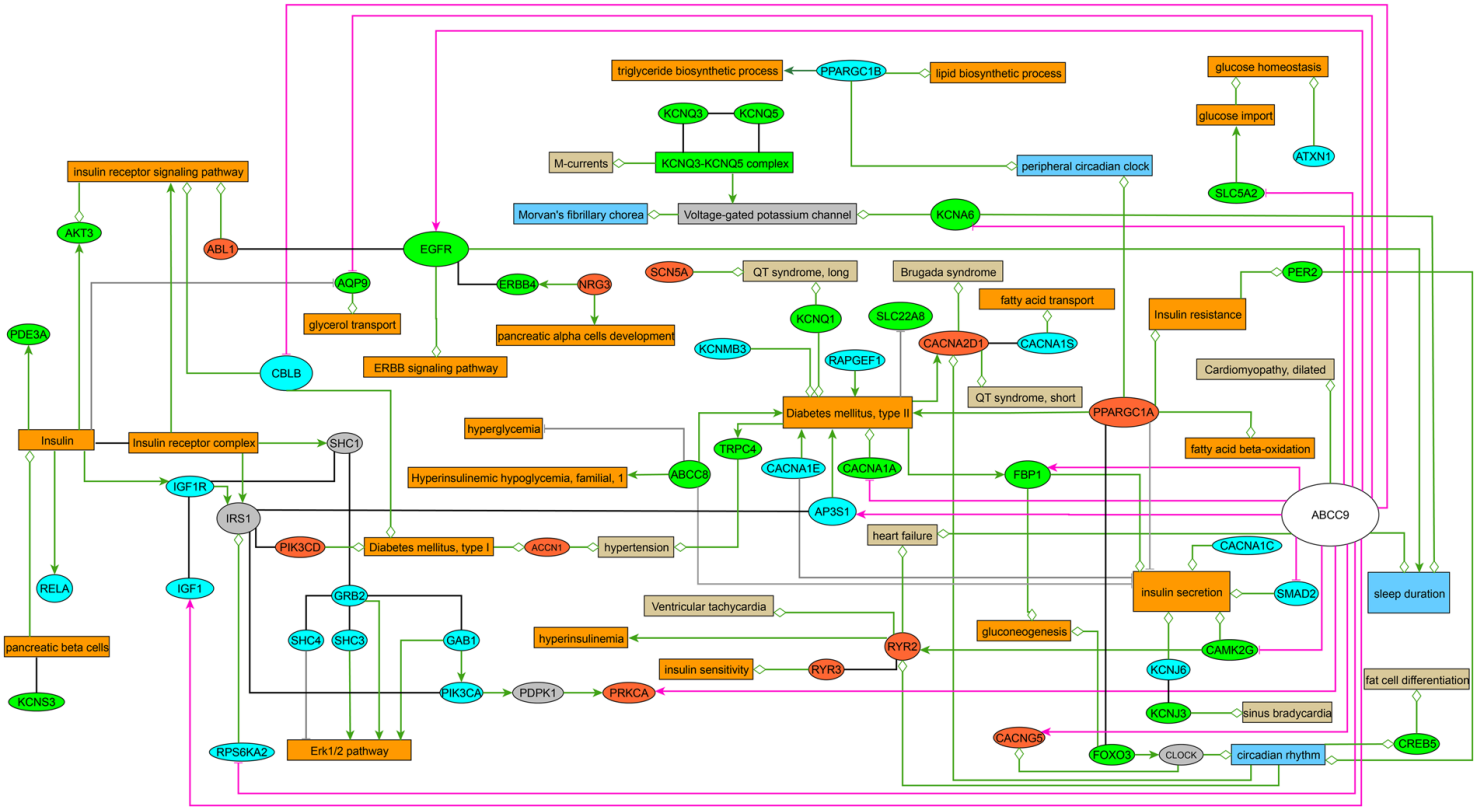
Human gene vs. disease multifactorial interaction network. The network shows interactions between genes from significant pathways (ERBB signaling family of tyrosine kinases and ion channels) identified based on the GWAS datasets (Meta3, green nodes; Meta7, light-blue nodes; overlapping genes between Meta3 and Meta7, orange nodes) and the Drosophila transcriptome GSEA. Only human homologs of the respective Drosophila genes with altered expression in the dSur KD flies in relation to controls (Fig. 2) were included. Relationships between the respective genes and biological processes (diabetes, carbohydrate and lipid metabolism, orange; cardiovascular diseases, beige), protein complexes and phenotypes (abnormal sleep and circadian behaviour, blue) are also shown (decreased activity/expression, grey edges with cross bar; increased activity/expression, green colored arrows; modulated activity/expression, green edges with open diamond). Red edges indicate gene expression for flies pooled every 3 h of the 24 hours period (decreasing expression, cross bar; increased expression in relation to wild-type controls, arrows; ratio RNAi/wt). Protein-protein interactions are displayed as black edges; interlinking genes are shown as grey nodes. A list of interactions with literature references is available in the Supplementary Table S8.

However, sleep is a complex trait arising from the accumulation of genetic effects acting within common biological pathways. Based on this principle, we have here used a novel approach for the interpretation of our GWAS data. In standard GWAS, the combined effect of weaker SNPs/genes that could be associated with a trait is ignored, and thereby, it is difficult to explore biological function and mechanism from a systems point of view^8^. To overcome this limitation of the method, we used a gene set enrichment approach to identify the main gene sets/pathways in our genome-wide association results for sleep duration. To explore the functional relevance of these pathways for sleep duration, we investigated gene expression alterations in a *Drosophila* short sleeping model^5^ (d*Sur* knockdown) *versus* control flies. Several studies suggest that many sleep pathways are conserved between flies and mammals^9^ and support the notion that studies in *Drosophila* give insights into the molecular basis of sleep in more complex organisms. Therefore, we used a systems biology approach to compare the results of gene expression in d*Sur* KD *Drosophila* with significant pathways from our GWAS (current and past)^5^ meta-analyses results (for study design see Supplementary Material and Methods, Fig. 1). Remarkably, the two main pathways associated with sleep duration in humans were ranking among the top significant pathways in our gene expression analysis in *Drosophila*. We also confirmed that the expression of several genes, reported to be involved in sleep regulation in model organisms, was down-regulated in the short-sleeping flies. The molecular pathways of these genes are also associated with human sleep duration. Moreover, several genes of the top raking GWAS and *Drosophila* transcriptome pathways are known to be involved in the development of metabolic dysfunction, as shown in Figure 1.

## Materials and Methods

### Study Populations

Data from 3 GWAS consisting of 1564 phenotyped and genotyped individuals (all of European ancestry and informative for sleep duration) were meta-analysed in the discovery phase (Table 1). A description of the cohorts included in individual GWAS on sleep duration, which are reported here for the first time (BREC – Brazilian Extreme Chronotypes, EGCUT – Estonian Genome Center, University of Tartu and GEC – German Extreme Chronotypes), can be found in Supplementary Materials and Methods (Supplementary Fig. S1; Supplementary Table S1 and Supplementary Text). The data of the GEC cohort was obtained through an online-based platform and biological samples were obtained by mailing participants self-collecting saliva sample-kits (Oragene; DNA Genotek, Inc., Ottawa, Ontario, Canada) for DNA extraction. The consistence of the method was tested comparing intra-individual blood and saliva DNA of a sub-sample of individuals (Supplementary Fig. S2). To replicate the results of our pathway analyses, we made use of independent meta-analysis results on sleep duration GWAS that we had published^5^. A description of the 7 cohorts included in that study (KORA – Cooperative health research in the Region of Augsburg, KORCULA – the Korcula study in Croatia, ERF – Erasmus Rucphen Family, EGCUT – Estonian Genome Center, University of Tartu, MICROS – the Micro-isolates in South Tyrol Study, NESDA – the Netherlands Study of Depression and Anxiety and the ORCADES – Orkney Complex Disease Study) can be found in the Supplementary Material and Methods (Supplementary Table S1) and in the original publication^5^.

**Table 1:** Meta-analysis top cluster-index SNPs (P < 10^−5^) for associations across all investigated autosomal chromosomes.

Chr	SNP ID (region)	Effect allele	CEU allele freq	Freq range	Genome-wide association studies (N) - Beta coefficients -				Meta- analysis *P*	Cluster SNPs (KB)	Locus (role), nearby *loci*	eQTL
					BREC (484)	EGCUT (749)	GEC (331)	Meta-analysis				
7	rs2299492 (126052682..126334114)	T	0.11	0.06–0.07	0.3672	0.305	0.4405	0.357	1.232e-06	19 (281)	*GRM8* (*Intron*)	*NA*
7	rs177580 (28465195..28473041)	C	0.44	0.40	NA	−0.252	−0.1663	−0.222	1.852e-06	4 (8)	*CREB5* (*Intron*)	rs12674890 YRI 9e-05
13	rs1414933 (39561593..39582022)	G	0.14	0.09–0.14	0.2657	0.248	0.2296	0.252	3.719e-06	10 (20)	*—*	*NA*
9	rs1336466 (26323942..26404780)	T	0.79	0.82–0.80	NA	−0.245	−0.2983	−0.2649	8.123e-06	2 (81)	—	*NA*
17	rs7210252 (72396626..72403826)	G	0.02	0.05–0.08	0.1104	0.479	0.3487	0.326	7.076e-06	4 (7)	*MGAT5B* (*Intron*)	rs276229 YRI 4e-05

Ind﻿ex SNPs from 5 different associated genomic regions are shown. All SNPs listed are in Hardy-Weinberg equilibrium (HWE, P > 0.05), Criteria for clustering SNPs were physical distance threshold of 250 KB in relation to the index SNP, the extent of LD with it (*r2* > 0.5), and association *P* < 0.01. ^b^The nearest reference locus is in bold typeface if the SNP cited is within it, and the role of the SNP is given between brackets. Reference *loci* are shown if any SNP of the cluster is located in a specific *locus* or within 20 KB up- or downstream of it. Chromosomes positions are based on the NCBI build 36. Total N for the meta-analysis was 1564, except for rs177580 and rs1336466 (N = 1080), for which results were not available for the BREC GWA. The effect (beta) was calculated based on either on the minor or the common allele, depending on the statistics packages used for the GWA analyses. eQTL = expression quantitative trait loci gene expression annotation from the SCAN database. Abbreviations: Chr = chromosome; MAF = minor allele frequency; SEM = standard error of the mean; NA = not available; BREC: Brazilian Extreme Chronotpyes; EGCUT = Estonian Genome Center, University of Tartu, and GEC: German Extreme Chronotypes.

### Ethical statement

All studies adhered to the tenets of the Declaration of Helsinki. All experimental protocols were approved by the local ethics committees in Brazil (at Hospital de Clinics of Porto Alegre and National Commission of Ethics and Research; Project 08-087 GPPG/HCPA, CONEP 15155: “Evaluation of the chronobiological profile of a Caucasian population from the Vale do Taquary – Rio Grande do Sul”), in Estonia (at University of Tartu; N° 234/T-12: “Omics for health: an integrated approach to understand and predict human disease”), and in Germany (at University of Munich; N° 170-08: “The genetics of the endogenous clock in humans”). Informed consents were obtained from all participants after explanation of the nature and possible consequences of the study.

### Phenotyping

Average weekly sleep duration (SD_av_) was assessed with the short version of the Munich ChronoType Questionnaire (MCTQ)^10, 11^. Sleep duration distribution was nearly normal in all cohorts, but to avoid any deviations of normality, the average weekly sleep duration (on work and free days) was normalised (Supplementary Material and Methods). Inclusion criteria were: (1) no use of an alarm clock on free days; (2) no shift-work during the last three months; and (3) no use of sleep medication (benzodiazepines and other pharmacological agents that influence sleep; see Supplementary Table S2).

### Genotyping and Imputation

Genotyping of BREC, EGCUT and GEC were accomplished all with the same platform (Illumina Human OmniExpress 700 K) at the Helmholtz Centre Munich or Estonian Biocentre Genotyping Facilities. The overall quality-control criteria excluded individuals with low call rates or deviation from Hardy-Weinberg equilibrium, excess heterozygosity, gender mismatch and bad clustering in the stratification analysis (Supplementary Fig. S3). Non-genotyped SNPs were imputed by using the 1000 Genomes reference panel and the IMPUTE2^12^ software. Cohorts included in the meta-analysed published GWAS^5^ were genotyped on a variety of platforms. The quality control and imputation details can be found in Supplementary Materials and Methods, Figure 4.

### Statistical Analysis

Genotypes consisting of both directly typed and imputed SNPs (N ≈ 4.9–9 Mio, as specified in Supplementary Table S3) entered the GWA analyses. To avoid over-inflation of test statistics due to population structure or relatedness, we applied genomic controls for the independent studies and meta-analysis (Supplementary Materials and Methods, Supplementary Fig. S4 and Supplementary Table S3). Linear regression (PLINK) for associations with normalized sleep duration was performed under an additive model, with SNP allele dosage as predictor and with age, age2, gender, normalized MSFsc (Midsleep on free days, corrected for sleep-depth accumulated during the workweek), season of assessment (dichotomized based on time of the year; day-light savings time - DST or standard zone time assessments) and BMI as covariates (Supplementary Fig. S5). A fixed-effects meta-analysis was conducted using the inverse-variance-weighted method in PLINK (Supplementary Fig. S6). All SNPs with low MAF (<0.01) and low imputation quality (Rsq/proper_info <0.3) were dropped from the meta-analysis. Corresponding to Bonferroni adjustment for one million independent tests^13^, we specified a threshold of *P* < 5 × 10^−8^ for genome-wide significance. To assess the number of independent loci associated with sleep duration at the *P* < 10^−5^ (Table 1 and Supplementary Table S4), correlated SNPs were grouped using a LD-based result clumping procedure (PLINK).

### Gene set enrichment analysis for GWAS

To identify pathways/gene sets associated with sleep duration, we used an improved gene set enrichment analysis for GWAS (i-GSEA4GWAS)^8^. This method overcomes the limitations of single SNP analysis in GWAS, using a gene set enrichment approach to yield pathways/gene sets associated with the trait of interest. Genes were mapped within 100 kb up- and downstream of the SNP (threshold *P* < 0.05) region. We searched for gene sets relevant for canonical pathways from a variety of online resources and GO biological processes, molecular function and cellular components. Pathways/gene sets with FDR < 0.25 were regarded as to be possibly associated with traits, whereas those with FDR < 0.05 were regarded as high confidence or with statistical significance (Supplementary Tables S5 and S6).

### Gene versus diseases network analyses

To integrate information of genes/proteins belonging to the most significant pathways with their validated relevance in several biological processes, we generated a multifactorial interaction network (Fig. 1). We used CIDeR, a publicly available database that integrates interactions between heterogeneous factors associated with human diseases^14^. All interactions shown in Figure 1 are based on experimental evidence from scientific literature (Supplementary Table S8: CIDeR interactions^14^). Our analyses covered disease-related interactions from sleep disorders, as well as the metabolic dysfunction (obesity and type I and II diabetes).

### Fly strains and Behavioural Assays

Flies were raised at 23 °C on yeast cornmeal agar food. An *elav*-*Gal4* strain was crossed to the RNAi *UAS*-*Sur* line (*V104241*) from the Vienna Drosophila RNAi Centre. The KD effect was quantified and found to be ~40% (Supplementary Fig. S7 and Supplementary Table S9). Control experiments were performed with *V10424* and *elav*-*Gal4* flies. *Drosophila* Activity Monitoring System devices (DAMS) from Trikinetics (Waltham, MA) were used to collect locomotor activity and sleep data in 1-min bins. Two-day-old males (32 flies per genotype) were acclimatised in 12 h:12 h light/dark cycles at 23 °C for two days, and activity was recorded under these conditions for 3 further days. Sleep deprivation was calculated from the locomotor activity data by using a Microsoft Excel script in which sleep was defined as 5 min of consecutive inactivity of the flies^15^. All animal procedures were approved by the local ethical committee at University of Padova and followed international guidelines.

### Microarray labelling and hybridization

Gene expression profiling was carried out on d*Sur* KD (elav-Gal4/RNAi-104241 knockdown) and parental control strain (RNAi-104241) fruit flies using the Drosophila 1.0 custom platform (Agilent Technologies) in two experimental conditions: “Pooled” (pooled samples collected every three hours over a 24 h period, including also ZT15) and “Night” (collected in the dark phase at ZT15 as representative of the time-point of pronounced sleep shortening in the mutant flies). Flies were collected from the incubator during darkness at the night time. Red light was used for a few seconds only to transfer the flies into vials prior to freezing. For each condition, “Pooled” and “Night”, total RNA was extracted from the head of 30 flies for each genotype, each biological replicate, and each time point. Four biological replicates were analysed for d*Sur* KD and control samples respectively for each time point for a total of 16 microarray experiments. Details on total RNA isolation procedure, microarray design and microarray labelling are presented in Supplementary Materials and Methods. Gene expression data are available in the GEO database with the accession number: GSE52764.

### Statistical analysis of gene expression data

Inter-array normalization of expression levels was performed with quantile method^16^ to correct possible experimental distortions. A normalization function was applied to the expression data of all the experiments and the values of within-arrays replicate spots were then averaged. Spot quality measures were done as described in Supplementary Material and Methods. Principal Cluster analysis, and profile similarity searches were performed with Multi Experiment Viewer version 4.8.1 (tMev) of the TM4 Microarray Software Suite^17^. The identification of differentially expressed mRNAs was performed with Linear Models for Microarray Data (LIMMA) program with default settings (FDR ≤ 5%)^18^. The normalized expression values of the biological replicates for each genotype were log2 transformed and averaged.

### Functional enrichment analysis of the transcriptomic’s results

Functional enrichment analysis of differentially expressed genes in the *dSur* KD relative to expression observed in the control flies in the “Night” (flies collected at ZT15) and “Pooled” (flies collected every 3 hours over a 24 hours period) experiments was performed using the Graphite web tool^19^, which is based on Reactome pathway database^20^. The hypergeometric test (Fisher Exact test), which estimates the chance probability of observing a given number of genes from a pathway among the selected differentially expressed genes, was applied. Raw *p*-*values* were adjusted using Benjamini-Hockberg method.

### Validation of relative gene expression by quantitative RT-PCR

**Figure 2:**
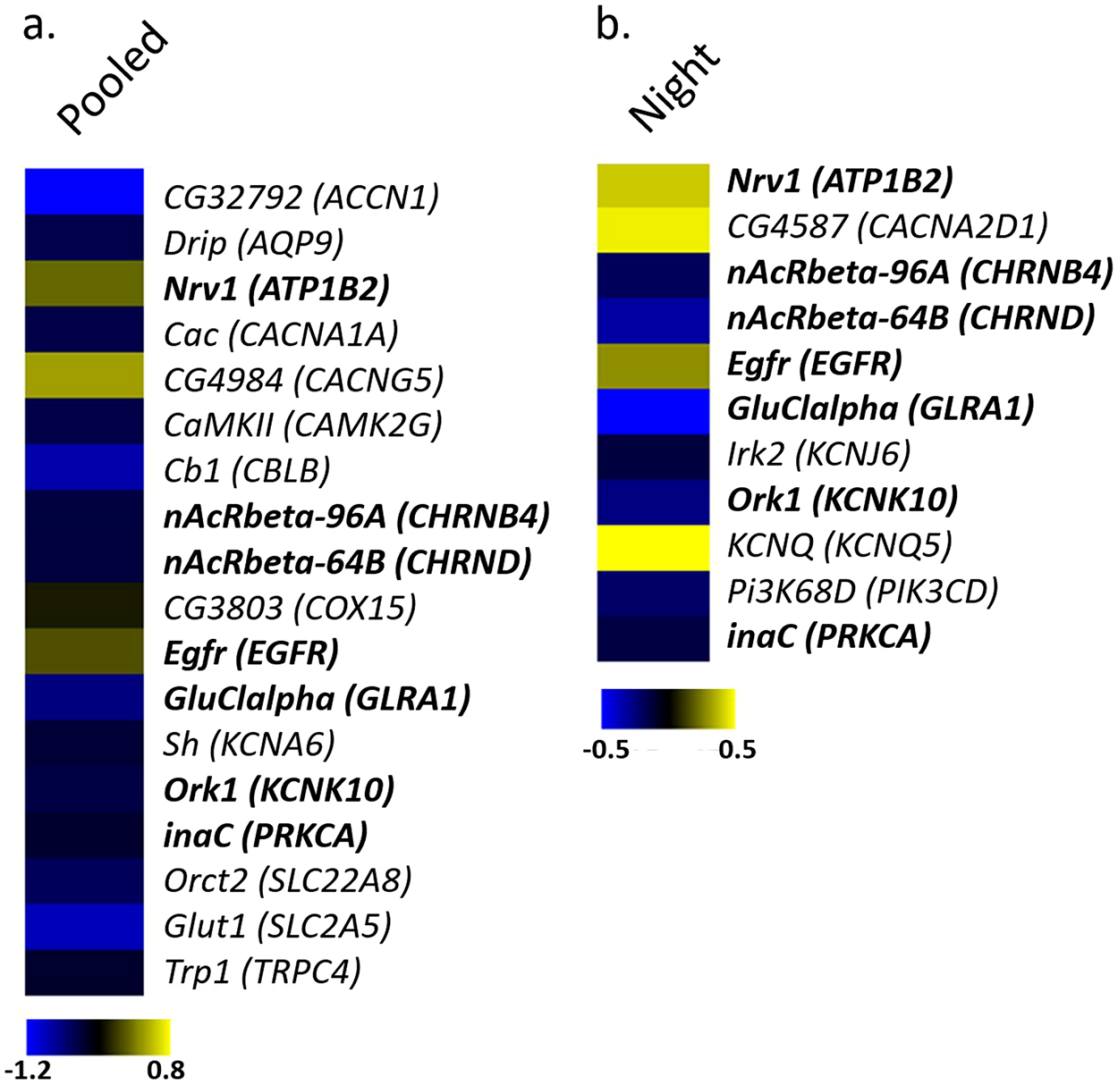
Differentially expressed genes in d*Sur* KD flies *vs*. controls. Heat map representing a selection of deregulated transcripts homologs to human genes (indicated in brackets) associated with sleep duration in the meta-analysis results used for the GSEA in (**a**) “Pooled” (*Drosophila* pooled every 3 h of the 24 hours period) and (**b**) “Night” (3 h into the night) conditions. A color-coded scale for the normalized expression values is used as follows: yellow and blue represent high and low expression levels in d*Sur* KD with respect to control, respectively. The expression level of each transcript was calculated as the log2 (d*Sur* KD/control), and the complete lists of differentially expressed genes identified by LIMMA software are provided in the Supplementary Table S7.

Quantitative RT-PCR was used to validate the expression values of four differentially expressed genes obtained from microarray experiments (Fig. 2). Experimental details can be found in the Supplementary Table S9 and in Supplementary Materials and Methods).

### Online tools

URLs: CIDeR, http://mips.helmholtz-muenchen.de/cider/; GaphiteWeb, http://graphiteweb.bio.unipd.it/index.html; LocusZoom, http://csg.sph.umich.edu/locuszoom/; i-GSEA4GWAS, http://gsea4gwas.psych.ac.cn/docs/documents.jsp; PLINK, http://pngu.mgh.harvard.edu/~purcell/plink/index.shtml.

## Results

Here, we report for the first time the GWAS data of our current meta-analysis on sleep duration (GEC, BREC and EGCUT; see Supplementary Figs S3–S6, S8 and Supplementary Tables S3 and S4). Following the traditional genomic analyses, no population stratification was observed after genomic control in any of the three investigated cohorts (inflation factors λ, 1.00 to 1.03 for the independent studies and 0.99 for the overall genome-wide meta-analysis), as shown on the quantile-quantile plots (Supplementary Fig. S4). Meta-analysis of all 3 studies (N = 1564) did not yield a genome-wide significant signal (Supplementary Fig. S6); while independent genome-wide significant hits were observed in the GEC cohort (Supplementary Fig. S5). A summary of the best ranking *loci* based on *P* values and number of correlated SNPs (see Materials and Methods for details) is listed in Table 1 (see also Supplementary Table S4 and Supplementary Fig. S8). We had shown earlier^5^ that the effect of *ABCC9* gene variant on epidemiological variation in human sleep duration is influenced by inter-individual differences both in chronotype (phase of entrainment) and seasonal differences in entrainment of the biological clock. We have therefore adjusted our analysis for season of assessment and chronotype. Studies not considering these confounding factors, may not be able^21^ to replicate our findings.

To get insights into the modulation of sleep duration by exploring the functional relationships among genes, we further analysed our current and past^5^ GWAS meta-analyses results to identify overrepresented gene-sets or pathways associated with sleep duration. Using a gene set enrichment analysis (GSEA) for the GWAS meta-analyses, we discovered a number of significant pathways (Table 2), including Circadian Exercise, a circadian pathway. This pathway was significant in both GSEA for the GWAS (Meta 7) and transcriptomic data (“Pooled” *Drosophila*), as shown in Table 2. Only two types of signalling pathways (ion channel genes and the ERBB signalling family of tyrosine kinases) associated with sleep duration in both GWAS meta-analyses and in the transcriptomic experiments; Table 2). We succeeded thereby to replicate these pathways, proving that this method can increase power for uncovering genetic factors relevant for sleep duration. As to be expected, only a few genes in these pathways were overlapping across studies (Fig. 1). To get insights into the functional relevance of these gene sets/pathways for sleep duration, we combined these findings to the results of transcriptome analysis from a short sleeping *Drosophila* model, knockdown for the *ABCC9* gene homolog (d*Sur*), *versus* control flies (Figs 1 and 2, experimental validation is presented in Supplementary Fig. S9). We performed the gene expression profiling of “Pooled” and “Night” d*Sur* KD since in these flies the quantity of sleep was affected both during the 24 h (22% of reduction) and at ZT15 (36% of reduction) as shown in Supplementary Fig. S9. Using LIMMA software with default settings (FDR ≤ 5%), we identified 4941 differentially expressed genes (DEGs), of which 2220 were up-regulated (45%) and 2721 were down-regulated (55%) and, 2885 DEG of which 1343 were up-regulated (47%) and 1542 were down-regulated (53%) in RNAi “Pooled” and “Night” samples with respect to controls (Supplementary Table S7). Functional enrichment analysis of DEGs in the *dSur* KD relative to expression observed in the control flies in the “Night” and “Pooled” experiments were conducted by using the Graphite web tool. The hypergeometric test (Fisher Exact test) was used to rank biological significance of differentially expressed genes in d*Sur* KD *versus* control flies in the “Night” and “Pooled” conditions. A list of all significant pathways can be found in the Supplementary Tables S5 and 6. Using this strategy, we confirmed that pathways replicated across GWAS meta-analyses were highly significant in the transcriptomic experiments (Table 2). Pathways that were significant only for one of the GWAS meta-analyses (in processes involving cell death, immuneresponse, signal transduction and metabolic processes), but had several corresponding significant pathways in the *Drosophila*, gene expression profiles comparisons are also shown in Table 2.

**Table 2:** Significant pathways in the independent GWAS meta-analyses and in the Drosophila transcriptomics study

i-GSEA4GWAS pathways	*Description*	Meta-analysis of 3 (7) GWAS			Transcriptomics *Sur* RNAi/control (Reactome pathways)	Night	Pooled
		*p*-*value*	FDR	Sig. genes/selected genes/all genes	Pathway	*q*-*value*	
**Substrate specific channel activity	Channel activity	**<0.001 (0,009)**	0.023 (0.188)	20/91/156 (20/87/156)	**Potassium Channels**	—	**0.007**
					**SLC-mediated transmembrane transport**	—	**0.007**
**Ion channel activity		**<0.001 (0,005)**	0.032 (0.147)	19/88/149 (20/85/149)	**Neuronal System**	**0.007**	**3.613 E-08**
					**Ion channel transport**	**0.032**	—
					**Potassium Channels**	—	**0.007**
*Calcium channel activity		**0.014 (0.010)**	0.146 (0.192)	7/24/33	—	—	—
hsa04620 toll like receptor signalling pathway	Cell death, imuno-response	NA (0.006)	NA (0.112)	NA (8/46/102)	Toll Like Receptor 4 (TLR4) Cascade	—	0.006
					Toll Like Receptor 10 (TLR10) Cascade	—	0.016
					Toll Like Receptor 5 (TLR5) Cascade	—	0.016
					Toll Like Receptor TLR1:TLR2 Cascade	—	0.016
					Toll Receptor Cascades	—	0.016
Immune response		NA (0.006)	NA (0.127)	NA (16/98/237)	Innate Immune System	—	5.845 E-05
G protein coupled receptor activity	Signal transduction, phosphorilation	<0.001 (NA)	0.033 (NA)	24/111/192 (NA)	G alpha (12/13) signalling events	0.017	0
					G-protein mediated events	0.011	<0.001
Neurotransmitter binding		0.002 (NA)	0.036 (NA)	6/30/53 (NA)	Transmission across Chemical Synapses	—	—
					Neurotransmitter Release Cycle	—	—
					Neurotrans. Receptor Binding/Downstream Transmission	—	—
Circadian exercise		0.004 (NA)	0.052 (NA)	5/20/47 (NA)	BMAL1:CLOCK/NPAS2 Circadian Expression	—	0.016
					Circadian Clock	—	0.049
Small GTPase mediated signal transduction		<0.001 (NA)	0.047 (NA)	9/44/90 (NA)	Downstream signal transduction	NA	**<0.001**
**hsa04012 ERBB signalling pathway		**0.013 (0.002)**	0.115 (0.074)	12/49/87 (13/46/87)	**Rho GTPase cycle**	**0.004**	**<0.001**
					**Signalling by Rho GTPases**	**0.004**	**<0.001**
					**Signalling by GPCR**	**0.012**	**2.181 E-08**
					**PLCG1 events in ERBB2 signalling**	—	**0**
					**Signalling by EGFR**	**—**	**0.006**
					**Signalling by ERBB2**	—	**0.061**
					**Signalling by ERBB4**	—	**0.065**
					**GPCR downstream signalling**	—	**3.815 E-07**
					**EGFR interacts with phospholipase C-gamma**	—	0
hsa04910 insulin signalling pathway	Metabolic processes	NA (0.021)	NA (0.196)	NA (10/59/135)	Regulation of Insulin Secretion	6.777 E-05	0.016
					Diabetes pathways	0.056	0.011
					Glucose metabolism	—	0.022
					Transport of glucose and other sugars, bile salts and organic acids, metal ions and amine compounds	—	0.022
					Glucagon signalling in metabolic regulation	—	0
					PKA activation in glucagon signalling	—	0
Nucleobase nucleoside and nucleotide metabolic process		0.001 (NA)	0.053 (NA)	5/22/52 (NA)	Amino acid and derivative metabolism	8.713 E-05	—
					Amino acid synthesis and transamination	<0.001	—
Nucleotide metabolic process		<0.001 (NA)	0.040 (NA)	5/21/42 (NA)	Nucleotide metabolism	0.001	0.001
hsa00240 pyrimidine metabolism		0.015 (NA)	0.129 (NA)	7/41/89 (NA)	Pyrimidine metabolism	0.004	—
Membrane lipid biosynthetic process		0.016 (NA)	0.137 (NA)	3/22/49 (NA)	Phospholipid metabolism	0.008	0.002
hsa00230 purine metabolism		0.025 (NA)	0.193 (NA)	14/71/145 (NA)	Purine ribonucleoside monophosphate biosynthesis	0	0
					Purine metabolism	0.030	0

iGSEA4GWAS detected pathways for the current and published GWAS meta-analyses results can be found at: http://gsea4gwas.psych.ac.cn/getResult.do?result=84441F977004419F41F381604BF24A36_1370614795252, and = 88D56D8FB785B71E515EFE87DD0FF064_1383051173051, respectively. Pathways/gene sets with false discovery rate (FDR < 0.25) are regarded as to be possibly associated with traits and with FDR < 0.05 are regarded as high confidence. A list of all significant pathways (q-value < = 0.05) for comparisons between d*Sur Drosophila* KD and control flies can be found at Supplementary Table S5 for flies collected in the night period and Supplementary Table S6 for pooled flies. *Pathways replicated in the independent meta-analyses; **Pathways that were both significant in the independent meta-analyses and in the *Drosophila* transcriptomic’s experiment. NA = not available, pathway was not significant or there was no genome coverage for trait associate; q-values = adjusted *p*-*values* with Benjamini & Hochberg method.

**Figure 3:**
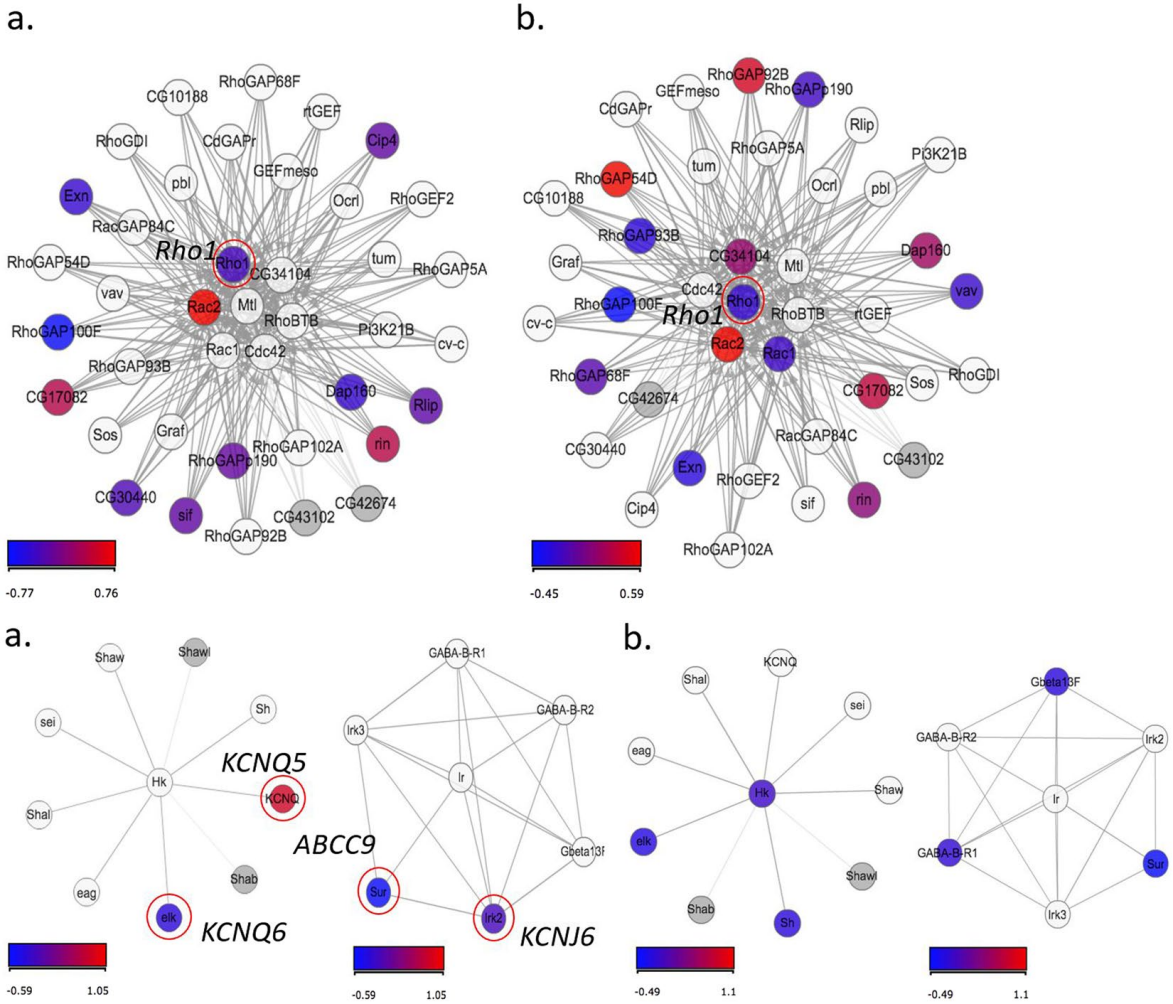
Signalling by Rho GTPases (upper panel) and Neuronal system pathway (bottom panel). These Reactome pathways show statistically significant enrichment (Fisher Exact test) of DEGs both in “Night” (**a**, upper panel left, *q*-*value* < 0.004; a, bottom panel left, *q*-*value* = 0.007) and “Pooled” (**b**, upper panel right, *q*-*value* < 0.001; b, bottom panel right, *q*-*value* < 0.001) conditions analysed with Graphite web tool. Coloured nodes represent the differentially expressed genes. The colour of the nodes is proportional to their expression levels represented as log2 (d*Sur* KD/control). Raw *p*-*values* were adjusted using Benjamini-Hockberg method (*q*-*value*).

To get a better idea of the network interactions among genes belonging to the replicated pathways in the human studies, we focused specifically on their respective *Drosophila* gene homologs (Table 2, Fig. 2). Here, we performed a multifactorial interaction networks analysis (Fig. 1) to connect these datasets to: (i) our data on differential gene expression in the d*Sur* KD vs. control flies (Fig. 2), (ii) information from the literature on gene-gene, and gene-protein interactions, and, (iii) published information on the relevance of these genes in the development of human diseases (Fig. 1, Supplementary Table S8). This approach yielded surprising evidences of the relevance of the GWAS associated pathways, which were significantly different in the *Drosophila* microarray experiments of the KD vs. control flies, *i*.*e*., the ERBB signalling pathway (Table 2 and Figs 2 and 3). Additionally, potassium channel genes from top ranking GWAS pathways were down regulated in the *Drosophila* short sleeping vs. control flies (Figs 1 and 2). Other top ranking pathways in humans and flies were related to insulin signalling and metabolism, with a down regulation of the genes involved in this pathway in the d*Sur* KD flies (Table 2, Fig. 2 and Supplementary Fig. S10). Differential expression of genes *Egfr* (*Epidermal growth factor receptor*), *InaC* (*inactivation no afterpotential C*), *Hk* (*Hyperkinetic*), and *Sh*-*isoF* (*Shaker isoform F*) was validated by qRT-PCR in both of the experimental conditions (night and pooled flies), as presented in Supplementary Materials and Methods, Supplementary Fig. S11 and Supplementary Table S9. Several other genes associated with T2D, involved in insulin secretion and lipid metabolism had differential expression in the d*Sur* KD vs. control flies and were nominally associated with sleep duration in humans (Figs 1 and 2).

## Discussion

Consistent with our previously published GWAS meta-analyses for sleep duration, the top ranking pathways identified both in human and *Drosophila* studies contained mostly ion channel genes, which are involved in various aspects of neural functions, immune responses, signal transduction (Figs 1 and 3, Table 2). Voltage-gated potassium channels (*e*.*g*., *KCNA6*- *Drosophila* shaker-related subfamily, member 6) identified in the top ranking pathways of our current meta-analysis in humans, was slightly down regulated in the d*Sur* KD vs. control flies (Table 2, Supplementary Fig. S11). Voltage-gated potassium channels genes were identified in *Drosophila*, and studies of genetic inactivation of their closest homologs in mammals proved their involvement in sleep duration also in mice^22^. However, in mammals there is a larger variety of genes encoding these channels, for instance, there is one *Shaker* gene in *Drosophila melanogaster*, but at least 16 genes code for α-subunits of voltage-dependent potassium channels in mammals^22^. Additional channels, such as *KCNQ* (*potassium voltage*-*gated channel subfamily Q*), which evolutionarily displays conserved electrophysiology and pharmacology between *Drosophila* and mammals^23^, had increased expression in the d*Sur* KD flies and 3 of the 5 *KCNQ* human genes appeared in the best ranking GWAS pathways (Table 2 and Fig. 2). Functional evidence of the relevance of these potassium voltage channels for sleep in humans comes from patients with a rare autoimmune disorder (Morvan’s syndrome; see cider multifactorial interaction network in human diseases, Fig. 1), who have marked sleeplessness associated with the presence of autoantibodies against voltage-dependent potassium channels^24^. A potassium ATP dependent channel (*KCNJ3*, *potassium voltage*-*gated channel subfamily J member 3*), which expression is inhibited in bipolar depression and schizophrenia, was down regulated in the Drosophila KD and the gene appeared as part of one of the significant pathways in the human GWAS (Table 2). Also voltage-gated calcium channels *CACNA2D1* (*auxiliary subunit alpha2delta 1*), *CACNG5* (*auxiliary subunit gamma 5*), *CACNA1S* (*subunit alpha1 S*), *CACNA1E* (*subunit alpha1 E*), *CACNA1C* (*subunit alpha1 C*) and *CACNA1A* (*subunit alpha1 A*) were among the best ranking gene sets in our pathway analyses in humans. One of them, *CACNA1A*, which encodes a subunit of *Cav2*.*1*, was characterized as a sleep gene in knockout mice^25^, showing reduced expression in our short-sleeping flies (Fig. 2). Related to these calcium channels, mutations in 3 of the genes present in the diagram *CACNA1C*, *CACNA2D1* (*calcium voltage*-*gated channel auxiliary subunit alpha2delta 1*) and *SCN5A* (*sodium voltage*-*gated channel alpha subunit 5*) have been shown to cause the *Brugada Syndrome*, characterized by typical ECG features, ventricular arrhythmias, which may be accompanied by sleep disordered breathing^26^.

### ERBB signalling pathway and sleep duration

For the first time the ERBB pathway, protein family of tyrosin kinases that consists of four cell surface receptors ErbB1/EGFR/HER1 (*epidermal growth factor receptor*), ERBB2/HER2 (*erb*-*b2 receptor tyrosine kinase 2*), ErbB3/HER3 (*erb*-*b2 receptor tyrosine kinase 3*), and ERBB4/HER4 (*erb*-*b2 receptor tyrosine kinase 4*)^27^, has been associated with sleep in humans. This is supported by robust evidence of the role of EGFR signalling pathway modulating sleep in *Drosophila* ^28^ and mammals^29^. EGF activates tyrosin kinases acting in intracellular signalling pathways, and in components of intracellular signal-transduction pathways^29^. *EGF* is expressed in the brain, influences the production of several sleep promoting substances^29^, and its exogenous administration in rabbits enhances non-rapid eye movement sleep. In our experiments, knocking down d*Sur* increased the expression of *EGFR* (Fig. 2) and shortened sleep duration in these flies in relation to wild type controls. In *Drosophila*, activation of EGF is dependent of Rho (also known as *Rhomboid*, *Rho1*, a Rho family GTPases, G protein-coupled receptors), and inhibition of Rho delays sleep consolidation in flies^28^. An increase in *EGFR* expression in our d*Sur* KD flies (Fig. 3 and Supplementary Fig. S11) can potentially be influencing sleep consolidation in a similar manner. *Rho* and *EGF* are expressed in the *pars intercerebralis*, a part of the fly brain that is functionally analogous to the hypothalamus in vertebrates, which is a region of the mammalian brain well established to be a regulator of arousal. The role of Rho in the presentation and intramembrane cleavage of TGF-α-like proteins in *Drosophila* suggests possible similar roles in the presentation of TGF-α-like proteins in vertebrates^30^. A *Drosophila Rho* homolog in humans (human rhomboid family 1, *RHBDF1*) was shown to interact with TGF-α family ligands^31^. Stimulation of EGFR signalling in human articular chondrocytes by TGFα results in the activation of RhoA/ROCK (Rho kinase), MEK (MAPK/ERK kinase)/ERK (extracellular-signal-regulated kinase), and PI3K (phosphoinositide 3-kinase) pathways. Our gene set enrichment analyses of genetic and transcriptomic data indicated that *EGFR*, *PI3K* and genes of the MAPK/ERK kinase pathway are linked to sleep shortening (Fig. 1). Moreover, *Rho* down regulation and *EGF* up-regulation in the d*Sur* KD link EGFR signalling pathway to the ABCC9 K_ATP_ potassium channel. Supporting this evidence, K_ATP_ channels, activated by pinacidil, triggered signalling through Rho kinase in mice^32^. *ABCC9* encodes a subunit of the KATP dependent channel which is crucial for cardiovascular and diabetic phenotypes^33^. EGFR inhibition prevents diabetes-induced up-regulation of multiple gene pathways in the mesenteric vasculature^34^. These results suggest that activation of EGFR signalling is a key initiating step that leads to induction of multiple signalling pathways in the development of diabetes-induced vascular dysfunction^34^. Our results support it, showing that knocking down the potassium channel, which leads to a diabetic/cardiomyopathies related phenotype in *Drosophila*, has downstream effects activating EGF expression. Moreover, they suggest that this is a shortening sleep duration pathway, thereby relating sleep regulation to key components of diabetes and cardiomyopathies canonical pathways. This is supported by the pathway regulating Insulin Secretion, which shows higher significance for flies collected 3 hours into the night (ZT15) than for the pooled flies experiment.

Interestingly, mammalian rhodopsin interacts with a Rho family member and activates this small GTPase in a light-dependent fashion^35^. However, no role has been described for transducing light activity through a rhodopsin/small GTPase pathway. Rhodopsin like receptor activity and a GPCRDB class, rhodopsin like, were significant pathways in the current meta-analysis and had similar pathways (GPCR signalling, Table 2) being altered in the transcriptomic study.

The notion that pathways modulating sleep duration could overlap between humans and flies was earlier laid by studies suggesting that the nervous system of both flies and mammals used similar arousal transmitters^9^. Various neurotransmitters act at cell-surface G protein–coupled receptors (GPCR, pathway which was also significant in our *Drosophila* gene expression profiles; Table 2) to activate intracellular signal transduction cascades. Activation of the G protein–coupled receptors (such as dopamine receptors) leads to an increase or decrease in adenylate cyclase, modulating cAMP levels. cAMP in turn activates protein kinase A (PKA), which phosphorylates a number of targets, including the transcription factor CREB (cAMP response element binding protein)^9^. The alpha catalytic subunits of PKA (*PRKCA* in humans, *InaC* in *Drosophila*) levels was increased in the d*Sur* KD (Fig. 2 and Supplementary Fig. S11) and the gene was detected in associated pathways of both sleep duration GWAS meta-analyses (Fig. 1). CREB activity is linked to sleep homeostasis as a CRE reporter gene is up-regulated in response to sleep deprivation and reduced CREB activity results in an elevated sleep rebound^9^. Supporting evidence from the GWAS could be found in our study for *CREB5* (*cAMP responsive element binding protein 5*) and *GRM8* (*glutamate metabotropic receptor 8*), which had the best association signals in the current GWAS meta-analysis (Table 1, Supplementary Fig. S8). *GRM8* is also part of the G protein coupled receptor activity pathway (Table 2), suggesting that although no genome-wide significance could be observed in single SNP analyses, we see the close connection of this to other genes within pathways that significantly associate with sleep duration (Fig. 1). Whereas only *CREB* has an established role in the transduction pathway, *CREB5* increases during sleep and decrease during sleep deprivation in mice (Supplementary Fig. S12).

Although the relationship between sleep duration and obesity may partially be caused by environmental influences such as voluntary sleep restriction and circadian misalignment, as indicated by previous epidemiological studies^36, 37^, the association of sleep duration with pathways related to energy metabolism indicates that genetic factors are central to it. A relation of sleep duration and metabolism was also demonstrated in *Drosophila*, as *cyc* ^*01*^ (canonical clock protein cycle) mutant flies can sustain periods of waking longer when starved than when sleep deprived, suggesting that the effect of sleep loss may be attenuated during starvation^38^. Gene expression analysis demonstrated that many of the genes with the greatest transcriptional differences between the sleep deprived and starved flies were genes involved with lipid metabolism suggesting a role in sleep homeostasis maintenance. Interestingly, down-regulation of genes involved in lipolysis and up-regulation of genes involved in triglycerides storage resulted in substantially increased sleep rebound following a night of sleep loss. In agreement with these observations, in the d*Sur* KD flies we observed a significant up-regulation of *bubblegum* (*bgm*, an acyl-CoA synthetase with homology to the human *SLC27a6*), *heimdall* (*hll*, a long-chain-fatty-acid–CoA ligase bubblegum-like) and *Lsd2* (*lipid storage droplet*-*2* ^39^) genes. These genes are involved in the control of lipid storage reducing lipolysis and blocking fatty acid release, and their *Drosophila* knockouts were shown to have sleep rebound alterations following sleep deprivation^38^. Upregulation of these genes in the d*Sur* KD can be thereby a consequence of sleep deprivation in our d*Sur* KD flies.

Our disease multifactorial interaction network shows that many of the genes from the pathways indicated to be relevant for sleep duration (Fig. 1, based on the human GWAS and *Drosophila* transcriptomics data), had functional evidence of their involvement with sleep regulation, circadian rhythms, insulin secretion, gluconeogenesis and lipogenesis. Moreover, genes that are key for energy metabolism were activated or inhibited in the short sleeping flies, potentially for playing a role on sleep shortening or responding to it.

## Electronic supplementary material

Supplementary Information

Supplementary Table S5

Supplementary Table S6

Supplementary Table S7

## References

[1] Allebrandt KV (2014). Chronotype and sleep duration: the influence of season of assessment. Chronobiol. Int..

[2] Wulff K, Gatti S, Wettstein JG, Foster RG (2010). Sleep and circadian rhythm disruption in psychiatric and neurodegenerative disease. Nat. Rev. Neurosci..

[3] Cappuccio FPF, D’Elia L, Strazzullo P, Miller MMA (2010). Sleep Duration and All-Cause Mortality: A Systematic Review and Meta-Analysis of Prospective Studies. Sleep.

[4] Laposky AD, Bass J, Kohsaka A, Turek FW (2008). Sleep and circadian rhythms: key components in the regulation of energy metabolism. FEBS Lett.

[5] Allebrandt KV (2013). A KATP channel gene effect on sleep duration: from genome-wide association studies to function in Drosophila. Mol. Psychiatry.

[6] Akrouh A, Halcomb SE, Nichols CG, Sala-Rabanal M (2009). Molecular biology of KATP channels and implications for health and disease. IUBMB Life.

[7] Maret S (2007). Homer1a is a core brain molecular correlate of sleep loss. Proc. Natl. Acad. Sci. USA.

[8] Zhang, K., Cui, S., Chang, S., Zhang, L. & Wang, J. i-GSEA4GWAS: A web server for identification of pathways/gene sets associated with traits by applying an improved gene set enrichment analysis to genome-wide association study. *Nucleic Acids Res*. **38** (2010). 10.1093/nar/gkq324 PMC2896119 20435672

[9] Kryger, M. H. b., Roth, T. d. e. & Dement, W. C. *Principles and Practice of Sleep Medicine*. *Principles and Practice of Sleep Medicine* (2005).

[10] Kantermann T, Juda M, Merrow M, Roenneberg T (2007). The Human Circadian Clock’s Seasonal Adjustment Is Disrupted by Daylight Saving Time. Curr. Biol..

[11] Allebrandt KV (2010). CLOCK Gene Variants Associate with Sleep Duration in Two Independent Populations. Biol. Psychiatry.

[12] Marchini J, Howie BN, Myers S, McVean G, Donnelly P (2007). A new multipoint method for genome-wide association studies by imputation of genotypes. Nat. Genet..

[13] Johnson RC (2010). Accounting for multiple comparisons in a genome-wide association study (GWAS). BMC Genomics.

[14] Lechner M (2012). CIDeR: multifactorial interaction networks in human diseases. Genome Biol..

[15] Shaw PJ (2000). Correlates of sleep and waking in Drosophila melanogaster. Science.

[16] Bolstad BM, Irizarry R, Astrand M, Speed TP (2003). A comparison of normalization methods for high density oligonucleotide array data based on variance and bias. Bioinformatics.

[17] Saeed A (2006). TM4 microarray software suit. Methods Enzymol..

[18] Gentleman, R., Carey, V., Huber, W., Irizarry, R. & Dudoit, S. Bioinformatics and Computational Biology Solutions Using R and Bioconductor. *Statistics for Biology and Health* (2005).

[19] Sales G, Calura E, Cavalieri D, Romualdi C (2012). graphite - a Bioconductor package to convert pathway topology to gene network. BMC Bioinformatics.

[20] Vastrik I (2007). Reactome: a knowledge base of biologic pathways and processes. Genome Biol..

[21] Gottlieb, D. J. *et al*. Novel loci associated with usual sleep duration: the CHARGE Consortium Genome-Wide Association Study. *Mol*. *Psychiatry* 1–8, doi:10.1038/mp.2014.133 (2014). 10.1038/mp.2014.133 PMC4430294 25469926

[22] Cirelli C (2009). The genetic and molecular regulation of sleep: from fruit flies to humans. Nat. Rev. Neurosci..

[23] Cavaliere, S. & Hodge, J. J. L. Drosophila KCNQ channel displays evolutionarily conserved electrophysiology and pharmacology with mammalian KCNQ channels. *PLoS One* **6** (2011). 10.1371/journal.pone.0023898 PMC3168433 21915266

[24] Liguori R (2001). Morvan’s syndrome: peripheral and central nervous system and cardiac involvement with antibodies to voltage-gated potassium channels. Brain.

[25] Deboer T, van Diepen HC, Ferrari MD, Van den Maagdenberg AMJM, Meijer JH (2013). Reduced sleep and low adenosinergic sensitivity in cacna1a R192Q mutant mice. Sleep.

[26] Macedo PG (2011). Sleep-disordered breathing in patients with the Brugada syndrome. Am J Cardiol.

[27] Citri A, Yarden Y (2006). EGF-ERBB signalling: towards the systems level. Nat. Rev. Mol. Cell Biol..

[28] Foltenyi K, Greenspan RJ, Newport JW (2007). Activation of EGFR and ERK by rhomboid signaling regulates the consolidation and maintenance of sleep in Drosophila. Nat. Neurosci..

[29] Kushikata T, Fang J, Chen Z, Wang Y, Krueger JM (1998). Epidermal growth factor enhances spontaneous sleep in rabbits. Am. J. Physiol..

[30] Bang AG, Kintner C (2000). Rhomboid and star facilitate presentation and processing of the Drosophila TGF-?? homolog Spitz. Genes Dev..

[31] Nakagawa T (2005). Characterization of a human rhomboid homolog, p100hRho/RHBDF1, which interacts with TGF-?? family ligands. Dev. Dyn..

[32] Sun H (2014). Label-free cell phenotypic profiling decodes the composition and signaling of an endogenous ATP-sensitive potassium channel. Sci. Rep..

[33] Nichols CG, Singh GK, Grange DK (2013). KATP channels and cardiovascular disease: Suddenly a syndrome. Circulation Research.

[34] Benter IF (2009). Early inhibition of EGFR signaling prevents diabetes-induced up-regulation of multiple gene pathways in the mesenteric vasculature. Vascul. Pharmacol.

[35] Balasubramanian N, Slepak VZ (2003). Light-mediated activation of Rac-1 in photoreceptor outer segments. Curr. Biol..

[36] Taheri S, Lin L, Austin D, Young T, Mignot E (2004). Short sleep duration is associated with reduced leptin, elevated ghrelin, and increased body mass index. PLoS Med.

[37] Roenneberg T, Allebrandt KV, Merrow M, Vetter C (2012). Social jetlag and obesity. Curr. Biol..

[38] Thimgan MS, Seugnet L, Turk J, Shaw PJ (2015). Identification of Genes Associated with Resilience/Vulnerability to Sleep Deprivation and Starvation in Drosophila. Sleep.

[39] Thimgan MS, Suzuki Y, Seugnet L, Gottschalk L, Shaw PJ (2010). The Perilipin homologue, Lipid storage droplet 2, regulates sleep homeostasis and prevents learning impairments following sleep loss. PLoS Biol..

