# Supplementary material for: Identifying pathways modulating sleep duration: from genomics to transcriptomics
Source: Sci Rep. 2017 Jul 4;7:4555. doi: 10.1038/s41598-017-04027-7 (PMC5496883; doi:10.1038/s41598-017-04027-7)
Supplement: Supplementary file 1 — Supplementary Information [file 41598_2017_4027_MOESM1_ESM.pdf]

Supplementary Information for

Identifying pathways modulating sleep duration: from genomics to transcriptomics

Karla V. Allebrandt[1*], Maris Teder-Laving[2], Paola Cusumano[3], Goar Frishman[4], Rosa Levandovski[5], Andreas Ruepp[4], Maria P. L. Hidalgo[5], Rodolfo Costa[3], Andres Metspalu[2], Till Roenneberg[1] and Cristiano De Pittà [3].

[1] Institute of Medical Psychology, Ludwig-Maximilians-University, Munich, Germany;
[2] Estonian Genome Center and Institute of Molecular and Cell Biology of University of Tartu, Estonian Biocentre, Tartu, Estonia;
[3] Department of Biology, University of Padova, Padova, Italy;
[4] Institute for Bioinformatics and Systems Biology (GmbH); Helmholtz Center Munich, German Research Center for Environmental Health, Neuherberg, Germany;
[5] Departamento de Psiquiatria e Medicina Legal, Universidade Federal do Rio Grande do Sul (UFRGS), Porto Alegre, Brazil.

*Correspondence to: Dr. Karla V. Allebrandt

Medical Faculty, Ludwig-Maximilians-University, Goethestr. 31. D-80336 Munich

E-mail: karlaviviani.allebrandt@gmail.com

This file includes:

Supplementary Figures: 12

Supplementary Tables: 9

Supplementary Materials and Methods

Acknowledgments

Supplementary References

SUPPLEMENTARY FIGURES AND TABLES

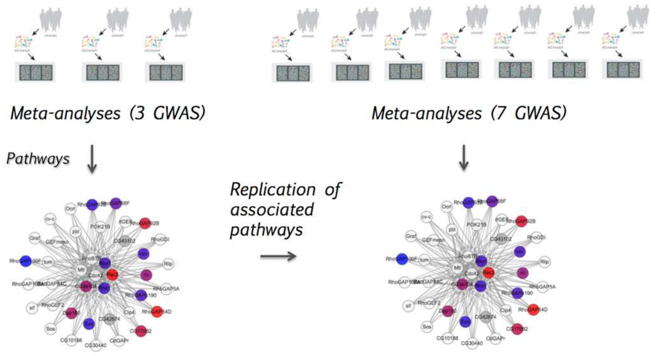

Figure S1. Study design. We conducted gene set enrichment analyses (GSEA4GWAS) by using the results of two independent meta-anlyses for sleep duration (first contained 3 GWAS reported in the current study and second contained GWAS in 7 populations reported previously[1]). With this method we identified significant pathways associated with sleep duration. Only two main kinds of signaling pathways (ion channel genes and the ERBB signalling family of tyrosin kinases) associated with sleep duration in both of the meta-analysis results used for the GSEA (Table 2). To visualize how the genes belonging to those pathways interact (botton pannel), we used a multifactorial interaction network, combining information from the literature and on gene expression of their homologs in a *Drosophila* short sleeping model. This multifactorial interaction network containing gene-gene, gene-protein interaction, and gene relation to disease development is presented in Figure 1 of the original manuscript.

WGGT of blood versus saliva DNA samples			
Samples	Concordant	Errors	Total
1	699738	1829	701567
2	724203	32	724235
3	589110	10475	599585
4	728299	25	728324
5	730008	17	730025
6	728030	24	728054
7	728136	47	728183
8	718739	112	718851
9	727608	20	727628
10	720866	239	721105

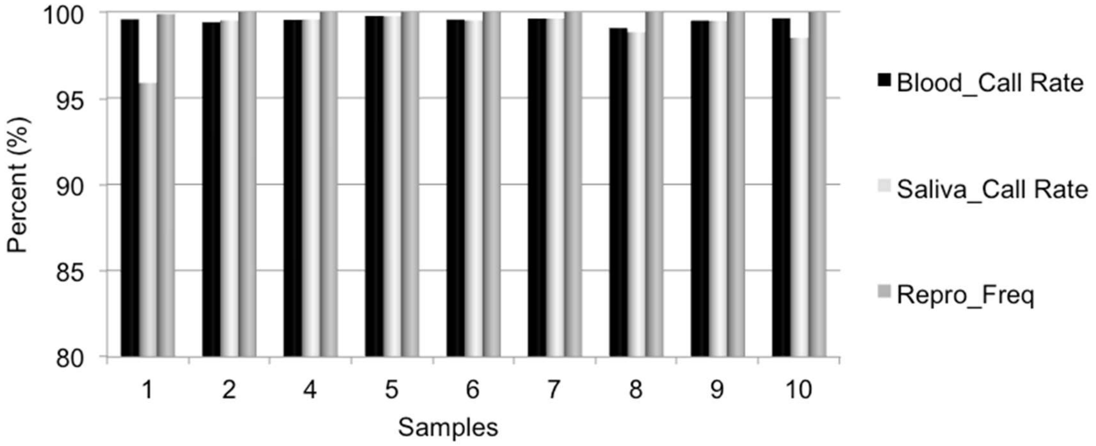

Figure S2. Whole-genome genotyping (WGGT) of intra-individual saliva and blood DNA samples. To test the reliability of the method, we evaluated here the concordance rates between 10 saliva- versus the respective individual blood-extracted DNA samples with the same high-density genotyping array (Illumina Human OmniExpress 700K). Genotypes were consistent across sources of biological material (upper Table); concordance rates between genotype calls of saliva- versus blood-extracted DNA samples from the same individuals were > 99.9 ± 3. Genotyping efficiency based on average genotype call rates (bottom panel) was similar for saliva- (97.3 ± 5%) and blood-extracted (99.5 ± 0.2%) DNA. Although blood-extracted DNA provides higher quality DNA, saliva-extracted DNA has proven to be a reliable method to obtain biological material for high-density genotyping arrays, being used in the GEC cohort.

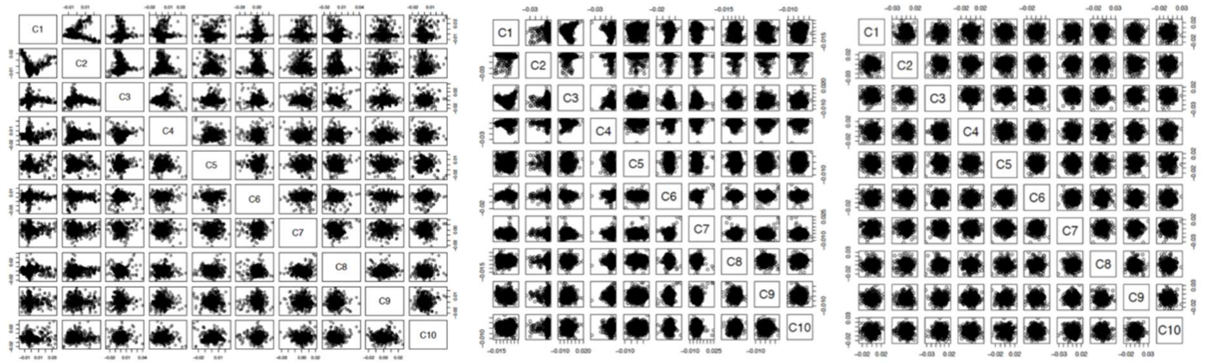

Figure S3. Multidimensional scaling plots showing components 1:10, which were used to adjust for population stratification in the analyses conducted with BREC (left panel), EGCUT (midle panel). GEC (right panel) did not present population stratification (inflation factor = 1) and association analyses were performed withouth adjustments for components.

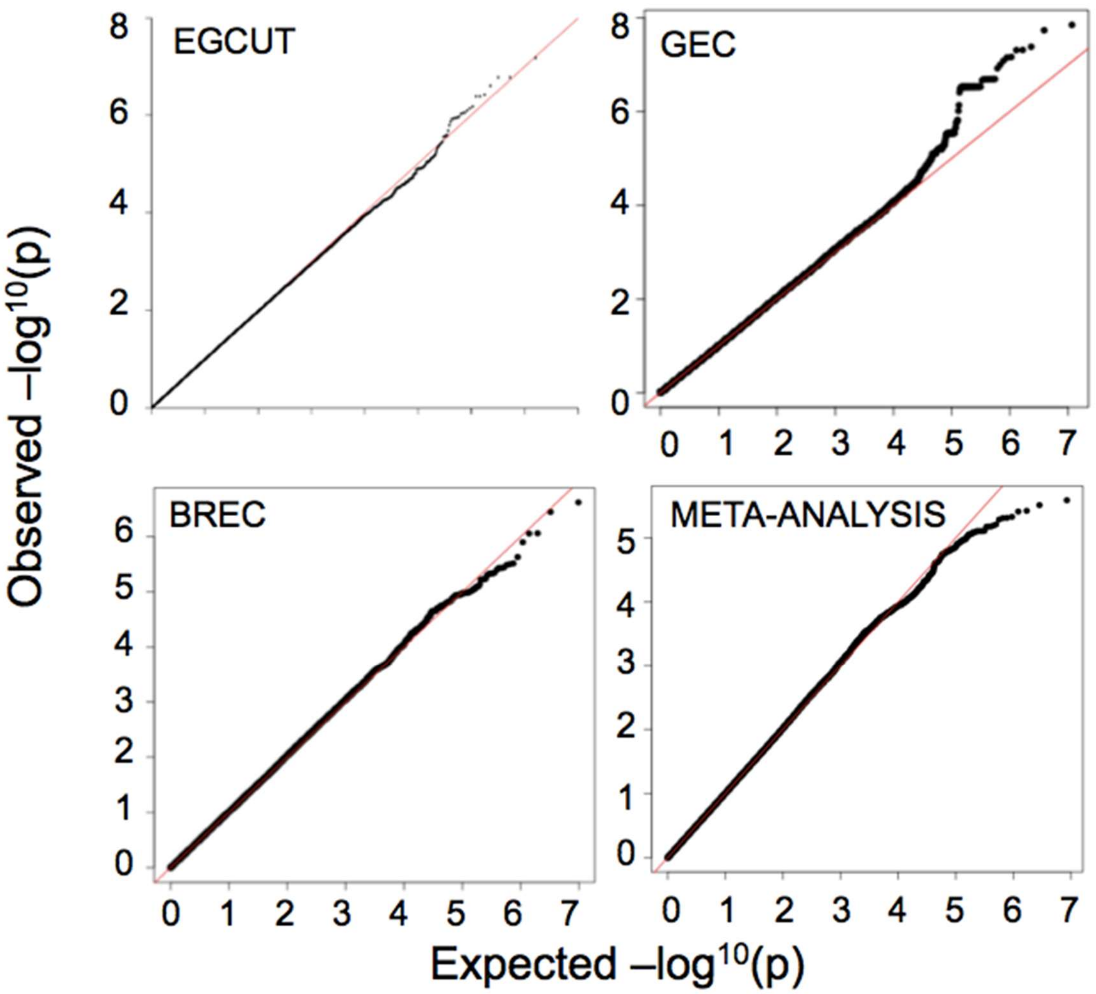

Figure S4. Study-specific and meta-analysis quantile-quantile plots. Quantile–quantile plots and lambda (λ) values were estimated for the primary analysis using the Cochran–Armitage trend test and for the confirmatory analyzes using logistic regressions and Cochran–Mantel–Haenszel stratified tests. Expected *P*-values are plotted against observed *P*-values resulting from each single study and for the meta-analysis after genomic control correction. All cohorts showed low over-dispersion of the chi-square statistics with the following λ-values: EGCUT = 1.02, BREC = 1.03, and GEC = 1.00. For the overall meta-analysis, the inflation factor (λ) was 0.99. If λ is large (for example, > 1.2), there is evidence that the observed test statistics deviate from the expected. This could be due to true associations but is more likely due to a systematic bias (for example, population stratification effects). Filled circles indicate observed association above expected (red line).

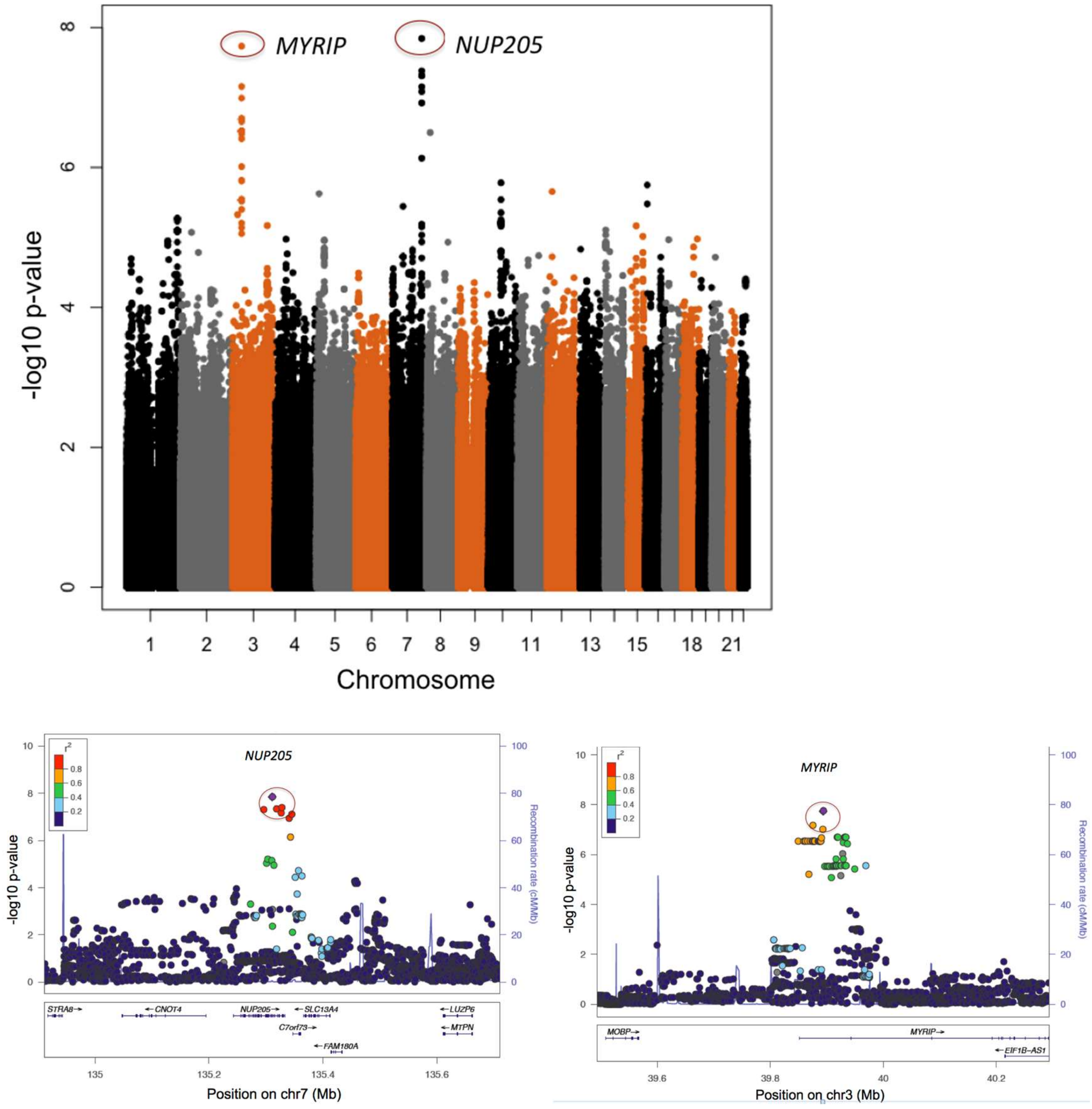

Figure S5. Manhattan plots of significant findings for GWAs reported for the first time in this manuscript. GEC (left upper panel) had two genome-wide significant hits (below the threshold of $P < 5 \times 10^{-8}$) in chromosomes 3 and 7, while BREC and EGCUT did not yield significant results (results no shown). Genomic locations with nearby genes are shown in the bottom panels. These results did not remain significant in the final meta-analyses.

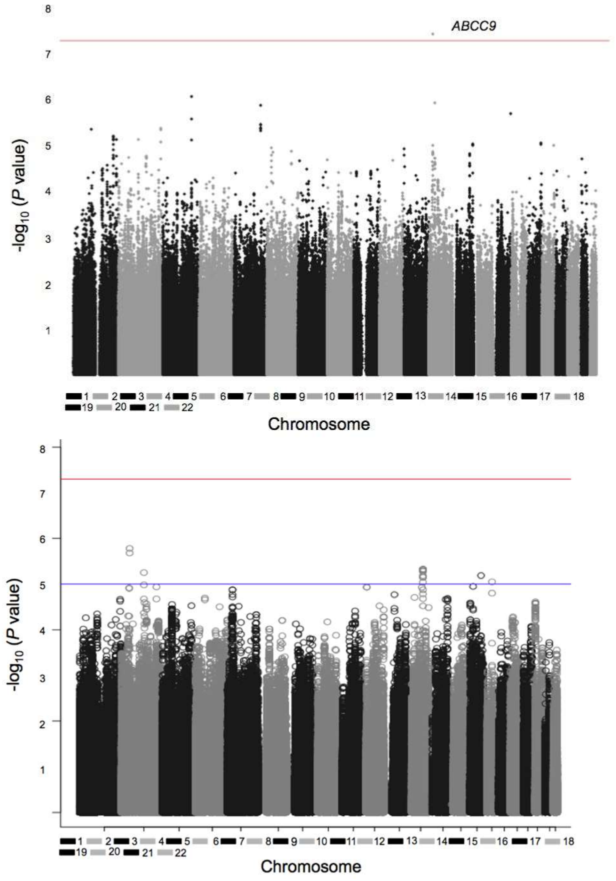

Figure S6. Manhattan plots of the meta-analysis results used in the pathway analysis. Left panel, meta-analyses of 7 cohorts[1] and, right panel, meta-analyses of 3 cohorts from GWAS data reported for the first time in this publication. SNPs are plotted on the x-axis according to their position on each chromosome against associations with sleep duration on the y-axis (shown as $-\log_{10} P$). The locus associated with sleep duration (rs11046205; $P < 5 \times 10^{-8}$) in our published meta-analysis is indicated (left panel). This SNP was not present in the datasets included in the current meta-analyses, which did not yield any genome-wide significant result (right panel).

a)

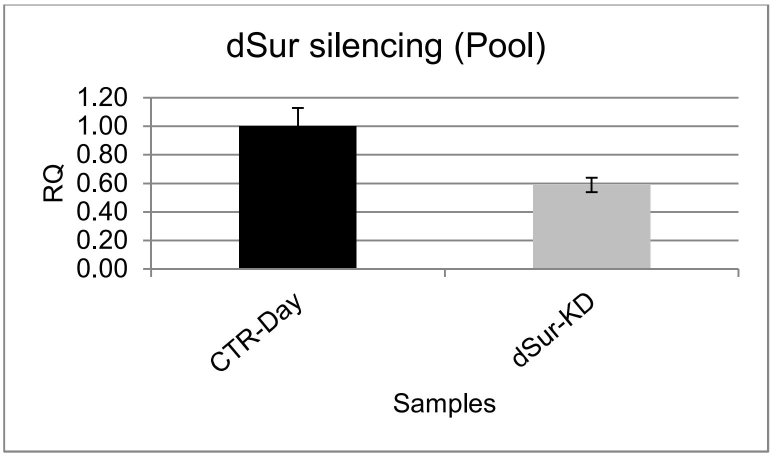

b)

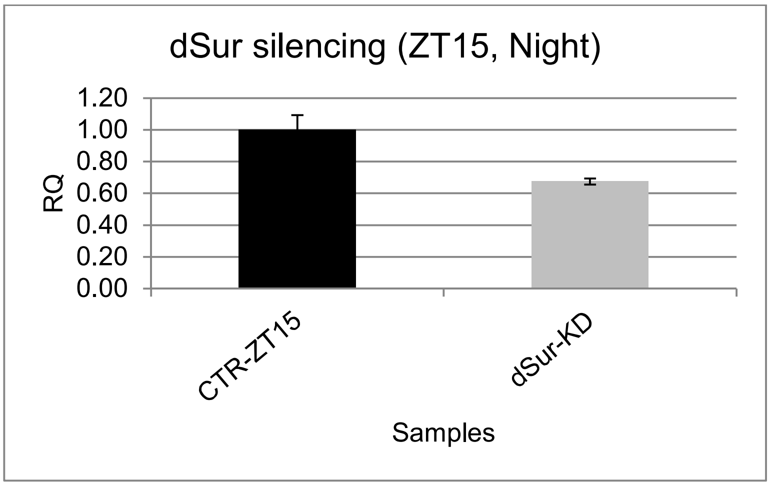

Figure S7. d*Sur* knockdown inhibition *versus* control (CTR) shown by qRT-PCR (QR) for both (A) "Pooled" and (B) "Night" conditions. (t-Test was performed: $p < 0.005$).

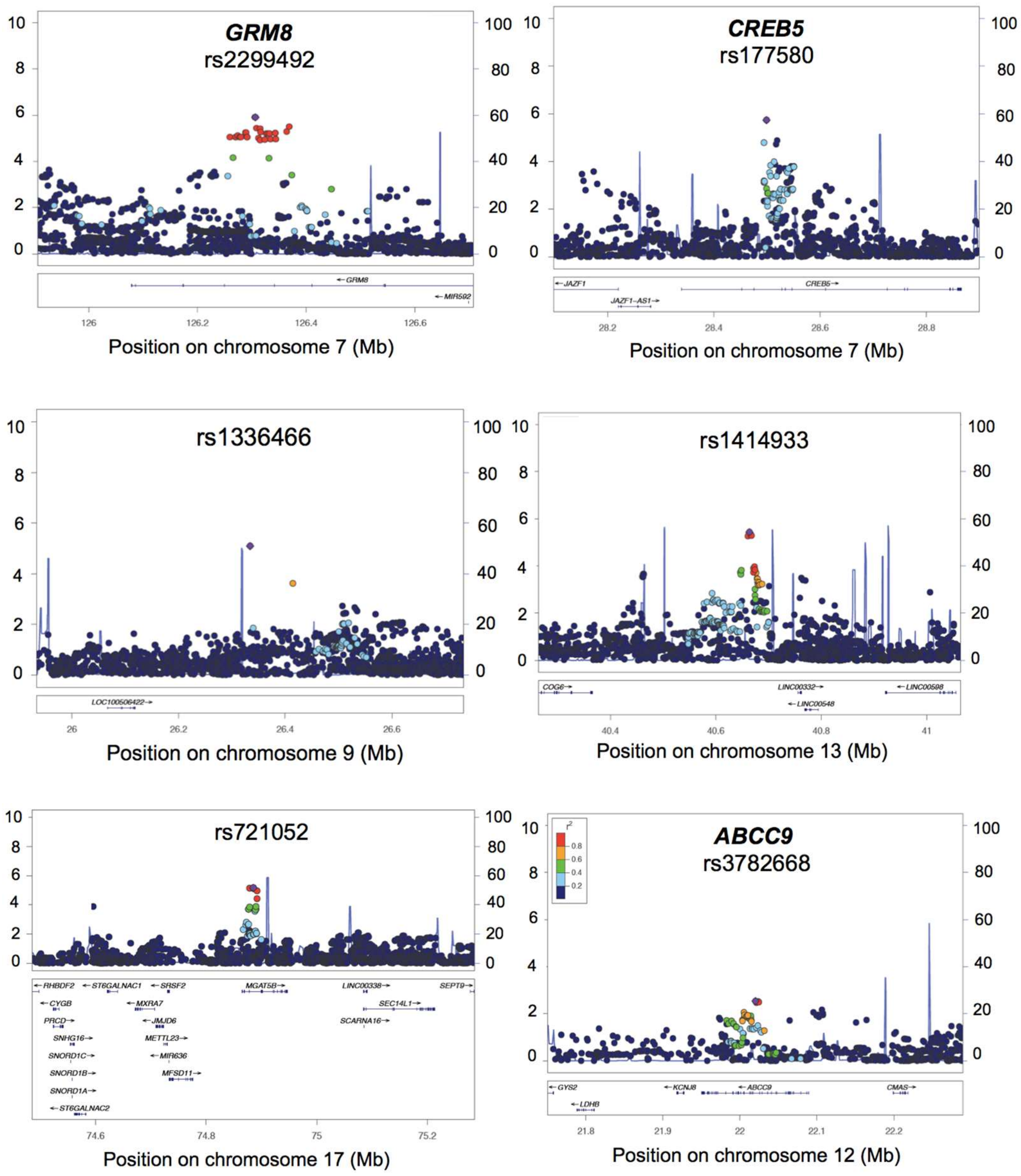

Figure S8. Genomic locations with nearby genes. Top 5 (top to bottom) associated genomic regions ($P < 10^{-5}$) as shown in Table 1 of the manuscript. The *ABCC9* gene region (bottom right) was enriched for signals with $P < 10^{-2}$. The *ABCC9* variant earlier associated with sleep duration (rs11046205) was not present in the current meta-analysis results and could not be imputed because of low linkage disequilibrium with surrounding variants.

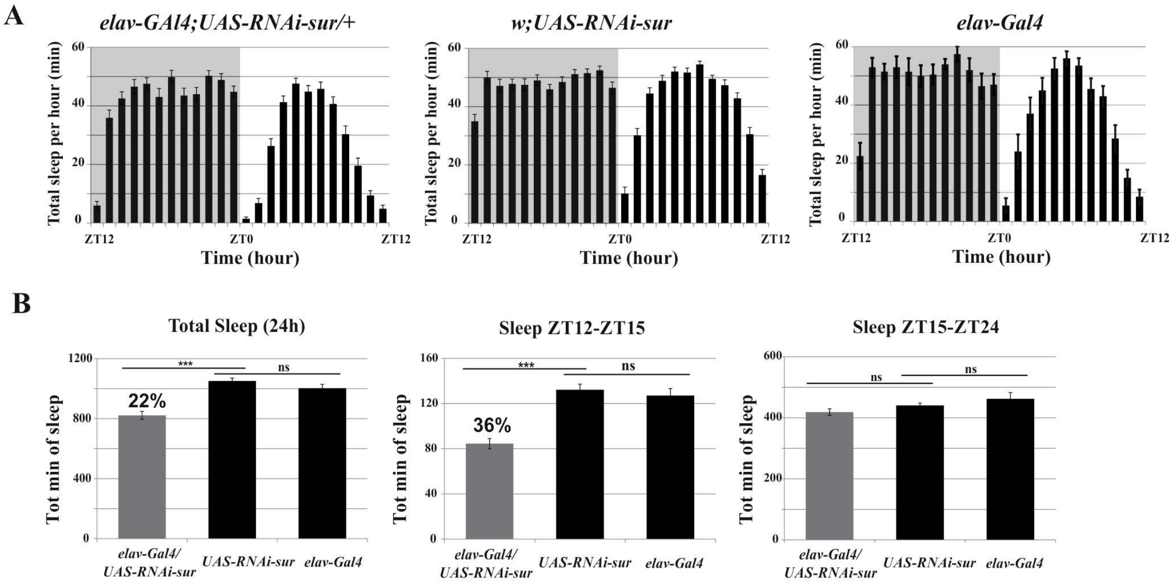

Figure S9. Pan-neuronal knockdown of d*Sur* in Drosophila decreases night sleep duration. The elav-Gal4 strain was crossed to the *UAS-Sur RNAi* line (V104241). (A) Plots of total sleep per hour (minutes) of KD and controls males in LD 12:12h light dark cycles over the 24 hours (grey background: night phase). (B) dSur KD flies show reduced sleep during the 24 hours day (22% of sleep reduction compared to controls) and in the first part of the night (ZT12-ZT15, 36% of reduction compared to controls. No effects on sleep amount was observed in the rest part of the night (ZT15-ZT24). (t-Test: *** $p < 0.005$).

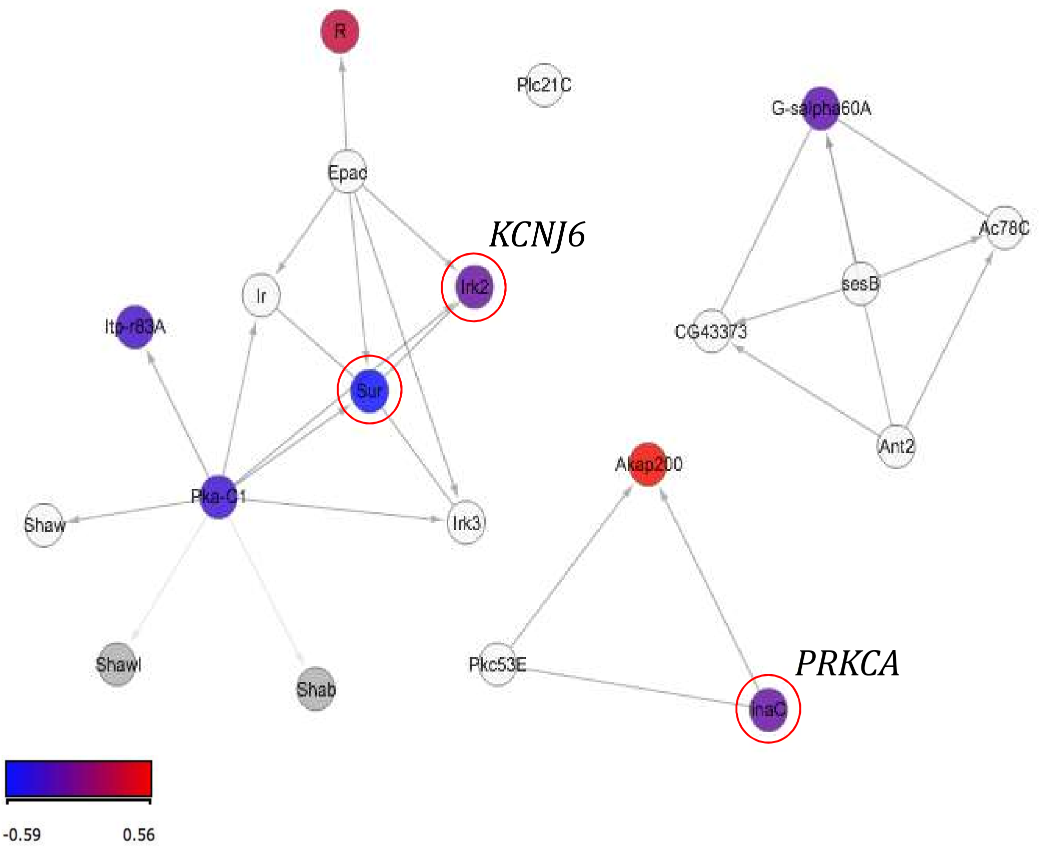

Figure S10. Insulin signalling pathway in *Drosophila*, with human gene homologs highlighted. This was a significant pathway (q-value < 0.05), identified with the Graphite web tool, from differentially expressed genes in the "Pooled" (q-value = 0.01) and "Night" (q-value < 6.7 x 10^{-5}) conditions.

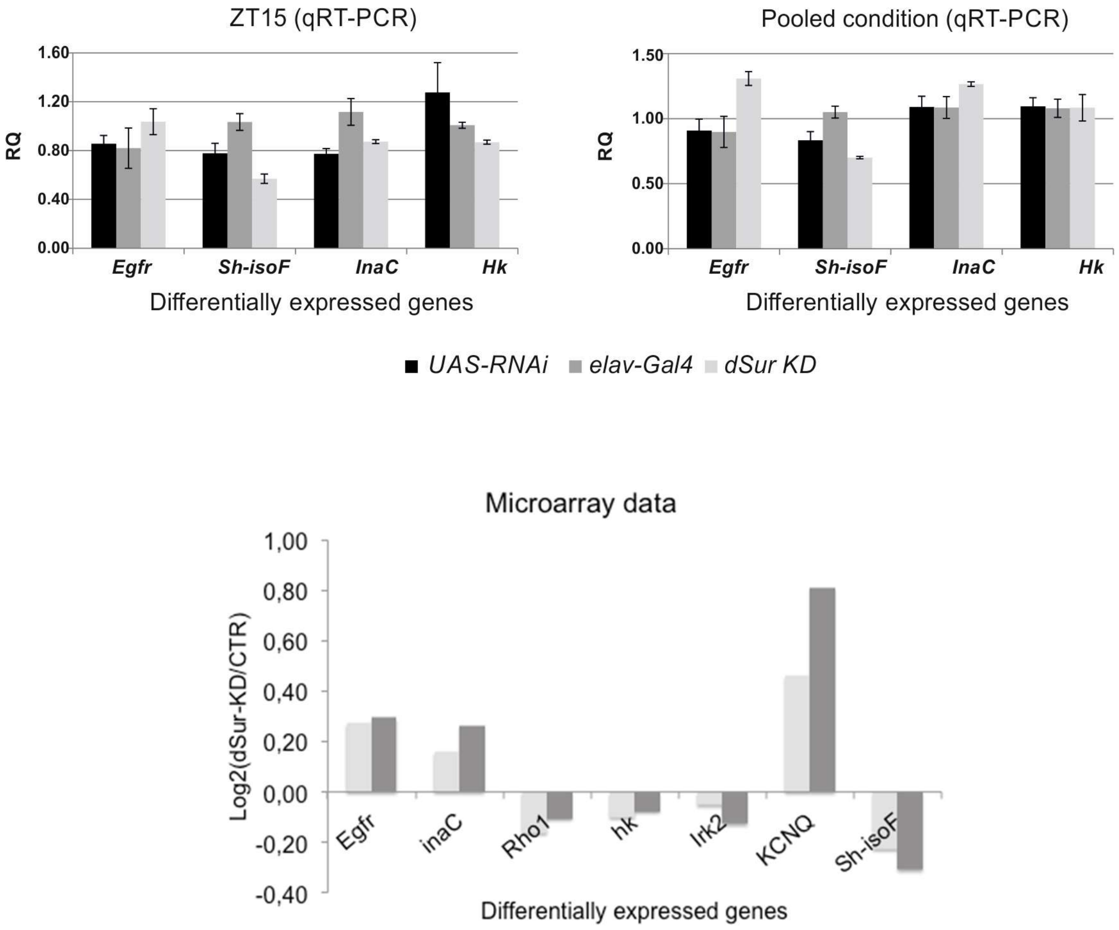

Figure S11. Validation of microarray expression values by qRT-PCR. (Upper panels) qRT-PCR expression levels defined in dSur KD vs. controls sampled in "Pooled" condition (Upper right panel) and at ZT15 "Night" condition (Upper left panel). For each condition, data are presented as the mean of relative quantity of template in the sample ± S.D. from three different biological replicates. T-test was performed. *Sh-isoF* (ZT15): *UAS-RNAi vs. dSur KD* *< p 0.05 and *elav-Gal4 vs. dSur KD* ***< p 0.005; *Egfr* (pooled): *UAS-RNAi vs. dSur KD* ***< *p* 0.005 and *elav-Gal4 vs. dSur KD* *< p 0.05. The bottom panel shows microarray expression levels defined in *dSur KD vs.* controls sampled in "Pooled" (light grey) and "Night" (dark grey) conditions. The expression level of each transcript was calculated as the log2 (*dSur KD / UAS-RNAi*).

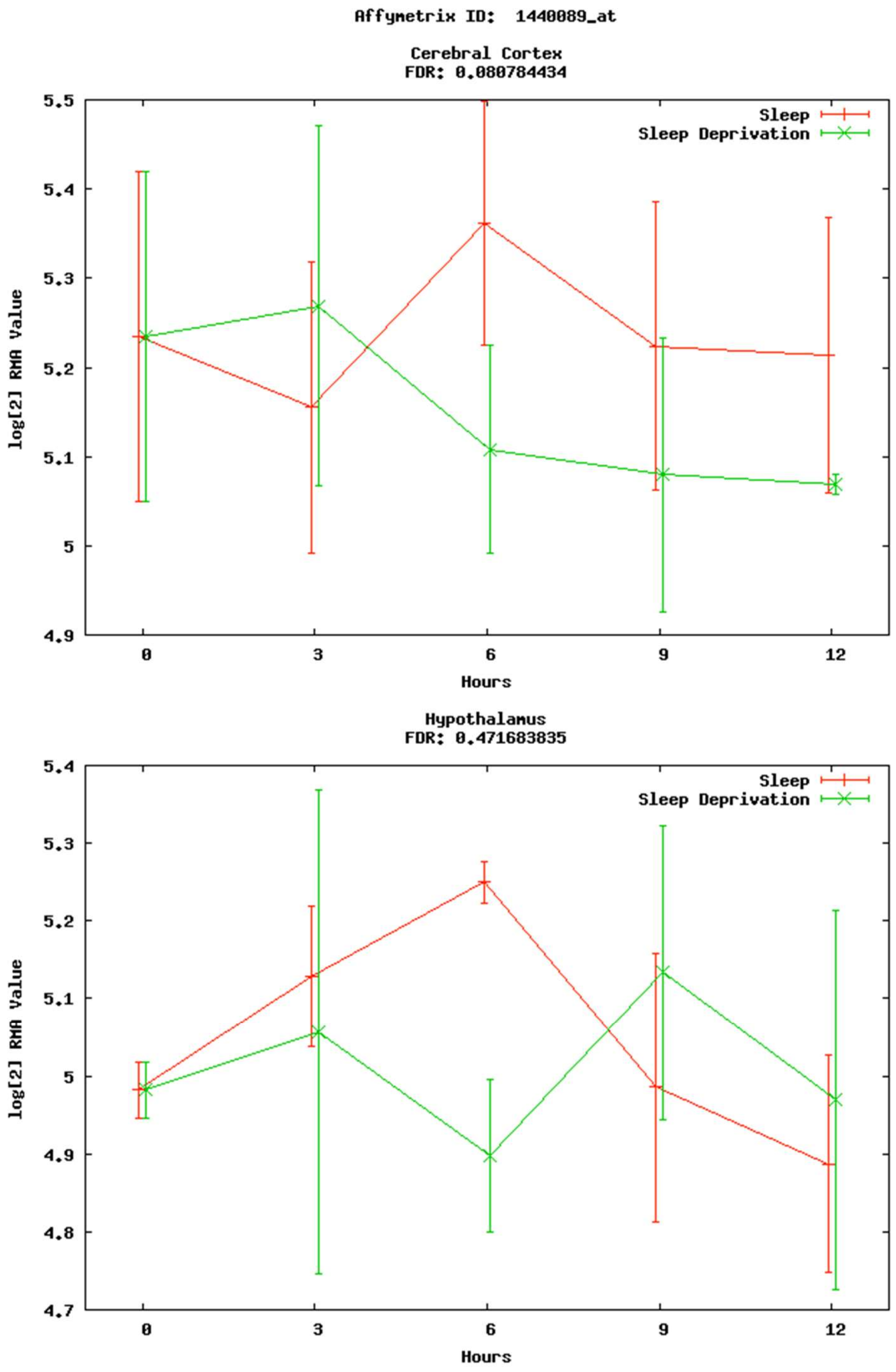

Figure S12. Creb5 increases during sleep and decrease during sleep deprivation in mice. Mouse Gene Expression Data. Souce: Sleep Gene: http://www.sleepgene.org/ResearchData/Mouse/Search.aspx.

Table S1. Study sample characteristics. Sample size, age, BMI, and sleep duration means per gender are shown by cohort for BREC, EGCUT, GEC from the current study and EGCUT, ERF, KORA F4, KORCULA, MICROS, NESDA, ORCADES from our earlier published GWAs.[1]

Data set (Ethnicity)	Sample size		Mean age y. (range)		Mean BMI ± SD		Mean sleep duration ± SD	
	total	females (males)	females	males	females	males	females	males
**BREC (German)**	484	334 (150)	43.5 (19 - 65)	46.7 (19 - 65)	25.8 ± 4.9	26.3 ± 4.1	7.81 ± 1.23	7.78 ± 1.43
**EGCUT (Estonian)**	750	343(407)	39.4(18 - 70)	39.4 (18 - 70)	25.8 ± 5.9	25.9 ± 4.5	7.80 ± 1.28	7.87 ± 1.19
**GEC (German)**	331	190 (143)	36.6 (18 - 70)	39.2 (18 – 70)	24.7 ± 5.6	26.1 ± 4.8	7.29 ± 1.01	7.0 ± 0.99
**Total**	1565							
Cohorts included in the meta-analyses from our earlier published GWAs.[1]								
**EGCUT (Estonian)**	924	518 (406)	41.0 (18 - 86)	38.2 (18 - 82)	25.6 ± 5.8	25.7 ± 4.6	7.92 ± 1.11	7.83 ± 1.14
**ERF (Dutch)**	740	397 (343)	46.7 (19 - 75)	50.0 (20 - 75)	25.8 ± 4.69	27.3 ± 4.21	7.57 ± 1.10	7.26 ± 0.95
**KORA F4 (German)**	548	279 (269)	54.6 (40 – 65)	55.3 (36 – 66)	26.6 ± 0.87	27.8 ± 4.21	7.30 ± 0.87	7.20 ± 0.81
**KORCULA (Croatian)**	600	383 (217)	55.6 (18 - 75)	57.3 (22 - 76)	27.8 ± 4.37	28.8 ± 3.56	7.34 ± 1.25	7.41 ± 1.14
**MICROS (Italian)**	693	374 (319)	39.3 (10 - 75)	41.1 (10 - 75)	24.2 ± 4.73	25.6 ± 4.04	8.20 ± 0.83	7.90 ± 1.07
**NESDA (Dutch)**	540	358 (182)	39.8 (18 - 66)	44.2 (19 - 65)	24.8 ± 5.00	26.0 ± 4.50	7.50 ± 0.90	7.80 ± 1.00
**ORCADES (Scottish)**	206	116 (90)	49.8 (18 - 73)	50.0 (17 - 71)	26.8 ± 5.13	26.7 ± 3.48	7.54 ± 0.88	7.26 ± 0.88
**Total**	4251							

Table S2. Pharmacological sleep agents. Drug groups and correspondent ATC codes used as exclusion criteria when selecting phenotyped subjects for the independent GWA studies [a].

Drug groups	Pharmacological ATC codes
Benzodiazepines	N05CD, N05CF
Barbiturates	N01AF, N01AG, N03AA, N05CA, N05CB, N05CX
Imipramine	N06AA02, N06AA03, N06AA06
Nortriptyline	N06AA10
Neuroleptics	N05AK
Phenothiazines	N05AB, N05AC, N05AA
Fluoxetine	N06AB03
Sertraline	N06AB06
Paroxetine	N06AB05
ß-Blockers, propranolol	C07, S01ED
Theophylline	R03DA04
Amphetamine	N06B

[a] With exception of the NESDA cohort, that only excluded subjects using benzodiazepines

Table S3. Stage-1 genotyping and imputation details across the different platforms used.

Populations	Genotyping Platform	Quality control of genotyped SNPs				Genetic Imputations software	NCBI Build	Analysis software	Total of SNPs for imputation	Total of SNPs in the meta-analysis	% of SNPs imput
		HWE *P*	CR SNP	CR IND	MAF						
BREC	OmniExpress 700K	10^{-5}	98%	99%	0.01	IMPUTE 2.0	37	PLINK	648 080	4 874 049	86.7
EGCUT	OmniExpress 700K	10^{-6}	95%	95%	0.01	IMPUTE 2.0	37	SNPTEST	609 547	9 249 616	93.8
GEC	OmniExpress 700K	10^{-5}	97%	99%	0.01	IMPUTE 2.0	37	PLINK	627 822	6 055 183	89.6

All individual association studies results underwent genomic control before meta-analysis. Cohorts were comprised of unrelated individuals. CR: call rate; MAF: minor allele frequency.

Table S4. List of best 100 associations with SD_{av} in our new meta-analyses. The file included Chromosome, genomic position, SNP rs codes, alleles, number of studies included in the meta-analysis, *P*-values and effect (beta).

CHR	Genomic Position	SNP	A1	A2	N	P	P.R.	Beta
13	42278410	rs28583896	C	T	2	1.227e-06	1.227e-06	-0.8936
7	126305306	rs116998487	C	A	3	1.232e-06	1.232e-06	0.3571
7	126306495	rs2299492	C	T	3	1.232e-06	1.232e-06	0.3571
7	28498869	rs177583	C	T	2	1.838e-06	1.838e-06	-0.2224
7	28498670	rs177580	C	T	2	1.852e-06	1.852e-06	-0.2224
1	164547894	rs56217471	C	G	3	1.877e-06	1.877e-06	-0.5955
7	126369228	rs2237765	G	C	3	3.113e-06	3.113e-06	0.3443
7	126308312	rs13243112	A	C	3	3.65e-06	3.65e-06	0.3373
13	40663593	rs1414933	G	A	3	3.719e-06	3.719e-06	0.2519
7	126315771	rs4731321	A	G	3	3.848e-06	3.848e-06	0.3427
2	196073314	rs12614225	G	A	2	4.585e-06	4.585e-06	0.6756
6	134093851	rs148960800	T	C	3	4.651e-06	4.651e-06	0.5168
13	40666492	rs7985465	G	C	3	5.073e-06	5.073e-06	0.2485
4	183014867	rs79698313	G	A	3	5.123e-06	0.03504	0.2257
13	40667486	rs2027203	T	C	3	5.283e-06	5.283e-06	0.2478
7	126364317	rs2237764	C	T	3	5.321e-06	5.321e-06	0.3386
10	105398890	rs148103957	C	A	2	5.375e-06	5.375e-06	0.9377
7	126316657	rs1815973	G	A	3	5.495e-06	5.495e-06	0.3371
13	40661167	rs9566494	G	A	3	5.534e-06	5.534e-06	0.2478
8	68285247	rs11780094	G	A	2	5.727e-06	5.727e-06	-0.2295
7	126288627	rs34055791	T	A	3	5.868e-06	5.868e-06	0.3409
7	126289582	rs2078808	A	C	3	5.868e-06	5.868e-06	0.3409
12	113485169	rs73205270	G	A	3	6.054e-06	0.01514	-0.2055
7	126343361	rs2283068	G	A	3	6.106e-06	6.106e-06	0.3363

7	126343421	rs2283069	G	T	3	6.106e-06	6.106e-06	0.3363
3	30618532	rs141391123	C	T	3	6.308e-06	6.308e-06	-0.6967
7	126326353	rs12667118	G	A	3	6.337e-06	6.337e-06	0.3357
7	126332301	rs3808153	T	A	3	6.387e-06	6.387e-06	0.3356
7	126329963	rs1008905	C	T	3	6.409e-06	6.409e-06	0.3355
12	113489545	rs55798985	G	A	3	6.509e-06	0.004619	-0.2051
4	170684647	chr4:170684647:D	GACTA	G	2	6.738e-06	6.738e-06	0.2156
3	30631726	rs186908119	C	T	3	6.85e-06	6.85e-06	-0.6883
17	74885031	rs7210252	G	T	3	7.076e-06	0.007705	0.3256
17	74885049	rs7211485	C	T	3	7.076e-06	0.007705	0.3256
17	74885176	rs7212262	A	G	3	7.09e-06	0.00773	0.3256
17	74878823	rs76943265	G	A	3	7.141e-06	0.007274	0.3266
17	74885595	rs7216131	C	T	3	7.147e-06	0.007851	0.3252
3	30647406	rs11466480	T	G	3	7.231e-06	7.231e-06	-0.6836
17	74885948	rs7216800	C	T	3	7.85e-06	0.008948	0.3235
7	126273998	rs13241983	T	C	3	7.857e-06	7.857e-06	0.3378
8	97647128	rs4734348	T	A	3	8.051e-06	0.03553	0.6493
9	26333942	rs1336466	T	C	2	8.123e-06	8.123e-06	-0.2649
3	95905630	chr3:95905630:D	TCTAA	T	2	8.661e-06	0.0001184	0.1793
15	100982234	rs2654593	G	A	2	8.861e-06	8.861e-06	0.2096
7	126270159	rs1419483	A	C	3	8.975e-06	8.975e-06	0.3353
7	126279831	rs35248039	A	C	3	8.975e-06	8.975e-06	0.3353
7	126260009	rs4728046	T	C	3	8.977e-06	8.977e-06	0.3353
7	126291048	rs77263654	C	A	3	9.005e-06	9.005e-06	0.3351
7	126291876	rs12706739	G	A	3	9.005e-06	9.005e-06	0.3351
7	126269978	rs1419482	T	C	3	9.136e-06	9.136e-06	0.3352
7	126278466	rs3808158	G	T	3	9.136e-06	9.136e-06	0.3352
3	95893870	chr3:95893870:I	C	CA	2	9.586e-06	9.144e-05	0.1788
15	57594556	rs10152152	A	G	2	9.933e-06	0.04066	-0.3901

1	157553577	rs75638380	C	T	2	9.948e-06	5.014e-05	-0.6264
2	196095297	rs138696578	A	C	2	9.982e-06	0.002369	-0.5985
7	135342916	rs10279223	G	T	2	9.997e-06	0.09644	0.2193
7	126312223	rs1579214	G	A	3	1.012e-05	1.012e-05	0.3163
7	126343862	rs35009695	A	G	3	1.083e-05	1.083e-05	0.3231
17	74891573	rs7218860	G	A	3	1.09e-05	0.01012	0.3264
4	170701875	rs2133954	A	G	3	1.118e-05	0.002088	0.1864
7	126334725	rs3808152	G	A	3	1.129e-05	1.129e-05	0.3224
7	126324176	rs10279616	G	T	3	1.134e-05	1.134e-05	0.3222
4	170706770	rs4692751	G	A	3	1.185e-05	0.000725	0.1821
14	90740803	rs73316773	A	C	3	1.217e-05	1.217e-05	-0.2643
7	126315112	rs2896374	C	A	3	1.225e-05	1.225e-05	0.3187
4	170706947	rs12650742	G	A	3	1.245e-05	0.0007975	0.1817
4	170706960	rs12644857	C	A	3	1.245e-05	0.0007975	0.1817
12	113489410	rs11612707	C	T	3	1.26e-05	0.003933	-0.1987
3	78107128	rs2872373	C	A	3	1.267e-05	1.267e-05	-0.1655
14	90758305	rs73318621	G	A	3	1.292e-05	1.292e-05	-0.2639
20	49339595	rs231584	C	T	2	1.298e-05	1.298e-05	-0.5176
3	77344784	rs9848444	C	T	3	1.312e-05	1.312e-05	-0.2486
5	34867	rs4956952	G	A	2	1.326e-05	1.326e-05	0.2285
7	28518799	rs217511	C	T	2	1.341e-05	1.341e-05	0.207
8	55385525	rs112233799	C	T	2	1.354e-05	0.0001959	-0.5614
6	33090305	rs115793178	G	A	2	1.385e-05	1.385e-05	-0.2176
12	113485599	rs11614358	G	A	3	1.4e-05	0.008084	-0.1966
18	25653386	chr18:25653386:D	TG	T	2	1.402e-05	0.0001311	-0.492
15	57599707	rs72736018	C	A	2	1.426e-05	0.0139	-0.4388
18	25637621	chr18:25637621:D	ACT	A	2	1.456e-05	1.456e-05	-0.5824
10	6176166	rs4747886	C	T	2	1.492e-05	1.492e-05	-0.2112
6	33090336	rs116242773	G	A	2	1.511e-05	1.511e-05	-0.2159

12	85068498	rs75873422	G	T	3	1.521e-05	1.521e-05	-0.33
12	85068731	rs111667343	C	T	3	1.521e-05	1.521e-05	-0.33
8	131651727	rs13255815	T	C	3	1.575e-05	2.381e-05	-0.1627
11	86919145	rs301592	G	T	3	1.584e-05	1.584e-05	-0.3597
20	49338809	rs190161	G	A	2	1.629e-05	1.629e-05	-0.5153
20	49338233	rs846747	C	T	2	1.655e-05	1.655e-05	-0.5152
7	28493761	rs76194790	C	T	2	1.673e-05	1.673e-05	0.2291
20	49337952	rs846746	T	C	2	1.696e-05	1.696e-05	-0.5145
11	86916076	rs540188	G	A	3	1.744e-05	1.744e-05	-0.3575
16	87493201	rs11641226	C	T	2	1.76e-05	0.03322	0.2697
20	49336632	rs405926	A	C	2	1.779e-05	1.779e-05	-0.5135
4	170698024	rs6553458	C	T	3	1.783e-05	0.001965	0.1915
4	170707495	rs62344460	T	C	3	1.831e-05	0.0005349	0.1863
19	52398105	rs11879492	T	A	2	1.836e-05	1.836e-05	0.5953
5	10701251	rs2918395	A	G	2	1.843e-05	0.01161	-0.247
4	170709315	rs6828442	A	C	3	1.857e-05	0.0004998	0.1862
4	183010367	rs9312286	T	C	3	1.86e-05	0.03302	0.2121
4	170708294	rs28769080	A	G	3	1.899e-05	0.0004763	0.186

Table S5. List of all significant pathways (q-value < 0.05) identified with Graphite web tool from 2885 differentially expressed genes in "Night" condition. To be downloaded as a separate excel file.

Table S6. List of all significant pathways (q-value < 0.05) identified with Graphite web tool from 4941 differentially expressed genes in "Pooled" condition. To be downloaded as a separate excel file.

Table S7. List of differentially expressed genes in d*Sur* KD *vs.* control flies detected by LIMMA analysis (FDR < 5%) in the "Pooled" (4941) and "Night" (2885) conditions. The expression level of each transcript was calculated as the Log2(d*Sur* KD/wt). To be downloaded as a separate excel file.

Table S8. List of the interactions with literature references supporting the interaction network given in Fig. 1.

Subject	Relation type	Object	PubMed ID	CIDeR ID
ABCC8 (polymorphism)	decreases activity of	hyperglycemia	18599530	24219
ABCC8 (mutation)	increases activity of	Hyperinsulinemic hypoglycemia, familial, 1	21968738	24300
ABCC8 (polymorphism)	increases activity of	Diabetes mellitus, type II	21968738	24312
ABCC8 (polymorphism)	decreases activity of	insulin secretion	17259403	24318
ABCC9 (mutation)	affects activity of	Cardiomyopathy, dilated	15034580	45160
ABCC9 (mutation)	affects activity of	heart failure	15034580	45157
ABCC9 (polymorphism)	affects activity of	sleep duration	22105623	45162
ABL1	affects activity of	insulin receptor signaling pathway	19251035	45130
ACCN1	affects activity of	hypertension	20064394	45052
AP3S1 (polymorphism)	increases activity of	Diabetes mellitus, type II	20512086	52055
AQP9	affects activity of	glycerol transport	25008241	52410
ATXN1	affects expression of	glucose homeostasis	27466200	73862
CACNA1A (mutation)	affects activity of	Diabetes mellitus, type II	9702684	52418
CACNA1C	affects activity of	insulin secretion	18562674	52424
CACNA1E (polymorphism)	decreases activity of	insulin secretion	17934712	52426
CACNA1E (polymorphism)	increases activity of	Diabetes mellitus, type II	17934712	52425

CACNA2D1 (mutation)	affects activity of	QT syndrome, short	21383000	45062
CACNA2D1 (polymorphism)	affects activity of	Brugada syndrome	23414114	45061
CAMK2G	affects activity of	insulin secretion	24627477	52100
CAMK2G	increases phosphorylation of	RYR2	23516528	52427
CBLB	affects activity of	Diabetes mellitus, type I	18201552	73756
CBLB (mutation)	affects activity of	insulin receptor signaling pathway	23349502	45562
circadian rhythm	affects expression of	CACNA2D1	18156197	45059
circadian rhythm	affects expression of	RYR2	19776257	43181
CLOCK	affects activity of	circadian rhythm	20591414	43220
Diabetes mellitus, type II	increases expression of	TRPC4	19393642	45587
Diabetes mellitus, type II	decreases quantity of	SLC22A8	24801871	52419
Diabetes mellitus, type II	increases expression of	CACNA2D1	21216827	45063
Diabetes mellitus, type II	increases expression of	FBP1	20567778	40913
Diabetes mellitus, type I	affects expression of	ACCN1	23723009	45050
EGFR	affects activity of	ERBB signaling pathway	9247128	46009
EGFR	increases quantity of	sleep duration	17694052	46002
EGFR	interacts with	ABL1	16273093	45092
EGFR	interacts with	ERBB 4	10572067	45095
FBP1	affects activity of	gluconeogenesis	20567778	40916
FBP1	affects activity of	insulin secretion	20719858	52074

FOXO3	affects activity of	gluconeogenesis	24692138	52322
FOXO3	increases expression of	CLOCK	24856209	52078
FOXO3	interacts with	PPARGC1A	19324885	52324
GAB1	increases activity of	ERK1/2 pathway	17178724	73794
GAB1	increases phosphorylation of	PIK3CA	10842168	52122
glucose import	increases activity of	glucose homeostasis	8360145	73838
GRB2	increases activity of	ERK1/2 pathway	9126968	73757
GRB2	interacts with	GAB1	11314042	52123
GRB2	interacts with	SHC1	8995379	52116
GRB2	interacts with	SHC3	8995379	52118
GRB2	interacts with	SHC4	17452444	52485
IGF1	interacts with	IGF1R	17315038	52315
IGF1R	increases activity of	IRS1	21912508	26009
IGF1R	interacts with	SHC1	7541045	52318
Insulin	decreases expression of	AQP9	25008241	52406
Insulin	increases activity of	AKT3	9480839	49191
Insulin	increases activity of	IGF1R	21912508	26008
Insulin	increases activity of	PDE3A	8070584	52530
Insulin	increases activity of	RELA	OA Inflammation 2013 Jun 01;1(1):7.	52520
Insulin	interacts with	insulin receptor complex	19041393	5929
insulin receptor complex	increases activity of	insulin receptor signaling pathway	19041393	73819
Insulin receptor complex	increases phosphorylation of	IRS1	7651388	52114

insulin receptor complex	increases phosphorylation of	SHC1	9165038	52119
insulin receptor signaling pathway	affects activity of	AKT3	12663464	52049
insulin resistance	affects activity of	PER2	23738904	52374
IRS1	interacts with	AP3S1	9792713	52053
IRS1	interacts with	PIK3CD	10744689	52486
KCNA6	affects activity of	Voltage-gated potassium channel	2347305	45473
KCNA6 (mutation)	affects activity of	sleep duration	15858564	45945
KCNJ6	affects activity of	insulin secretion	7626127	51763
KCNMB3 (polymorphism)	affects activity of	Diabetes mellitus, type II	23826284	52422
KCNQ1 (mutation)	affects activity of	Diabetes mellitus, type II	23271129	45580
KCNQ1 (mutation)	affects activity of	QT syndrome, long	14642002	45578
KCNQ3	interacts with	KCNQ5	12890507	43195
KCNQ3	is part of	KCNQ3-KCNQ5 complex	12890507	43196
KCNQ3-KCNQ5 complex	affects activity of	M-currents	12890507	43200
KCNQ3-KCNQ5 complex	increases activity of	Voltage-gated potassium channel	12890507	43199
KCNQ5	is part of	KCNQ3-KCNQ5 complex	12890507	43197
KCNS3	is expressed in	pancreatic beta cells	14928843	52331
NPY	affects activity of	insulin secretion	23211512	52357
NRG3	increases activity of	ERBB4	9275162	45094
NRG3	increases activity of	pancreatic alpha cell development	23610133	45126

pancreatic beta cells	affects quantity of	Insulin	19817786	54464
PDPK1	increases quantity of	PRKCA	17617409	52110
PER2	affects activity of	circadian rhythm	22117616	61417
PIK3CA	increases activity of	PDPK1	23131847	52097
PIK3CA	interacts with	IRS1	23643389	73795
PIK3CD	affects activity of	Diabetes mellitus, type I	23039284	45124
PPARGC1A (polymorphism)	decreases activity of	insulin secretion	18270681	23643
PPARGC1A	affects activity of	insulin resistance	19770177	5104
PPARGC1A	affects activity of	peripheral circadian clock	21325336	52325
PPARGC1A	affects activity of	fatty acid oxidation	19770177	13131
PPARGC1A (mutation)	increases activity of	Diabetes mellitus, type II	12107756	5219
PPARGC1B	affects activity of	lipid biosynthetic process	19254570	5250
PPARGC1B	affects activity of	peripheral circadian clock	21325336	52326
PPARGC1B	increases activity of	triglyceride biosynthetic process	19770177	4953
RAPGEF1 (polymorphism)	increases activity of	Diabetes mellitus, type II	19297053	73820
RPS6KA2	affects phosphorylation of	IRS1	24036112	52373
RYR2	affects activity of	heart failure	16701909	45081
RYR2 (mutant)	increases activity of	hyperinsulinemia	23516528	52428
RYR2	interacts with	RYR3	12213830	43190
RYR2 (mutation)	affects activity of	Ventricular tachycardia	21768539	45079
RYR3	affects activity of	insulin sensitivity	23389954	52431
SHC3	increases activity of	ERK1/2 pathway	12598680	73758

SHC4	decreases activity of	ERK1/2 pathway	28213521	73759
SLC5A2	increases activity of	glucose import	25344694	60201
SMAD2	affects activity of	insulin secretion	24068386	52377
TRPC4	affects activity of	hypertension	24113457	45586
Voltage-gated potassium channel	affects activity of	Morvan's fibrillary chorea	23712055	43336

Table S9. Primers used in quantitative RT-PCR.

ID Gene	Forward Primer (5'-3')	Reverse Primer (5'-3')
dSur	ACGTGGACAATCCAAGTGAAC	CGAGTGTCCTCCGTCATGT
Egfr	AATCTCAGGGCGGATCTATG	ACTGATCCGTGCCAGCTC
InaC	TTTCTTCCGGAATGTGGACT	TTGCTTATATCCTTGCGATGTTT
Hk	CCTCGAATTGCCAGTGCT	TCACACTCATCTTCGGCTTG
Sh-isoF	CCCCACTCGCACTTTAAATAAT	GCCATTCGCTCATTTTCACT

SUPPLEMENTARY MATERIALS AND METHODS

Description of the cohorts included in the current GWAS

BREC: Participants were locals of 12 counties in the Taquari Valley (Brazil); European descents (Germans and Italians) who immigrated to this region between 1824 and 1870. From the initial 6505 participants, 4051 subjects (1340 men and 2711 women; mean age: 44.1 ± 13.4, ranging from 18 to 65 years) had valid informative for sleep duration and chronotype.[2] Individuals selected as tails from the age and sex adjusted chronotype distribution had their blood samples collected and DNA isolated. After quality control (of DNA for whole-genome genotyping and of genotypes, as well as stratification analyses; Supplementary Figure S3) a remaining sample of 484 subjects was included in the GWA analyses.

GEC: Participants were assessed through an online survey focusing on extremes of chornotype. Based on a databank of about 65 000 chronotyped individuals in Germany, we selected 5500 extreme chronotype to participate in the online survey that generated the current cohort. We selected 670 participants who fulfilled the inclusion criteria described in phenotyping method to participate in our GWAS study. We mailed to 489 of those participants self-collecting saliva sample-kits (Oragene; DNA Genotek, Inc.,Ottawa, Ontario, Canada) and received 463 saliva samples (95% of total). After DNA isolation and quality control criteria for whole-genome genotyping, 378 high-quality DNA samples were genotyped with an average call rate of 99.0 ± 3%. However, approximately 14% of the saliva-extracted DNA did not meet quality control criteria (inspection of absorbance scans and gel electrophoresis) even after using a re-purification protocol- and could not be used for WGGT. The genotype concordance between saliva and blood extracted DNA from 10 invidious was compared to validate the method (Supplementary Figure 2). After genotyping and stratification quality control, 331 individuals were included in the GWA analyses.

EGCUT: The Estonian Genome Center, University of Tartu (EGCUT) is a bio-bank consisting of data of *ca* 51800 individuals from a population based Estonian cohort aged 18 years and older (67% females).[3] For the current study, GWAS was performed on 749 subjects, genotyped (array according to Illumina protocol) and phenotyped (with the MCTQ questionnaire) in the Estonian Genome Center. The cohort consisted of individuals selected as tails from the age and sex adjusted chronotype distribution of 5098 individuals. Study sample characteristics are presented in Supplementary Table 1.

Phenotyping

To normalize the average sleep duration individual values for the phenotype were deducted from the mean of the population and divided by the standard deviation of the mean. In the same way, we have normalized the mid-sleep on free days (chronotype) to use it as a covariate in the association analysis with sleep duration.

Characterisation of Drosophila knockdown

The knockdown was quantified and found to be ~40% (Supplementary Figure 7 and Supplementary Table 9). The qRT-PCR reaction was performed using a GoTaq qPCR Master Mix (Promega) using one µl aliquot of 1:100 diluted first-strand cDNA following the manufacturer's instructions on 7500 Real-Time PCR System (Applied Biosystems). The *dSur* RNA was amplified with exon spanning primers (Forward: *acgtggacaatccaagtgaac,* Reverse: *cgagtgtcctccgtcatgt),* and RNA levels were calculated relative to the *RP49* housekeeping gene. PCR reactions were performed in triplicate. Thermal cycling conditions were as follows: 2 min denaturation at 95°C followed by 38 cycles for 25 sec denaturation at 95°C, 1 min annealing and elongation at 60°C, and a final 3 min elongation at 72°C. The $2^{-\Delta\Delta Ct}$ (RQ, relative quantification) method implemented in the 7500 Real Time PCR System software was used to

calculate the relative expression ratio.[4] 95% confidence intervals are associated to each time point.

Total RNA isolation

Flies were collected every 3 hours after 3 days of entrainment in 12h:12h light/dark cycles at 23°C. Total RNA was extracted from the head of 30 flies for each genotype (d*Sur* KD and parental control), at each time point (ZT0, ZT3, ZT6, ZT9, ZT12, ZT15, ZT18, ZT21) and for each biological replicate. Four biological replicates were analysed for both d*Sur* KD and control samples for each experimental condition: "Pooled" (ZT0, ZT3, ZT6, ZT9, ZT12, ZT15, ZT18, ZT21) and "Night" (ZT15) for a total of 16 microarray experiments. All RNA samples were checked for quality by capillary electrophoresis (RNA 6000 Nano LabChip, Agilent Bioanalyzer 2100, Agilent Technologies). Only total RNA samples with R.I.N. (RNA Integrity Number) values higher than 7 were used for microarray analysis.

DNA microarray design

Probes were designed using the Agilent eArray Custom Microarray Design Service (https://earray.chem.agilent.com/earray/index.jsp), which applies proprietary prediction algorithms to design 60 mer oligo-probes. Microarrays were synthesized *in situ* using the Agilent ink-jet technology with 8 x 60 K format. A total of 32,162 probes representing *D. melanogaster* transcripts were successfully obtained. A custom microarray platform, named "*Drosophila* 1.0" (eArray Design ID: 035757), showed 30,814 duplicate probes and 1348 single probes. Each array included default positive (1,011 probes) and negative (308 probes) controls. Probe sequences and other details on the microarray platform can be found in the Gene Expression Omnibus (GEO) database (http://www.ncbi.nlm.nih.gov/geo/) under accession number: GPL17290.

Microarray labeling and hybridization

800 ng of total RNA was labeled with "Agilent One-Color Microarray-Based Gene Expression protocol" according to the manufacturer's instructions. The synthesized cDNA was transcribed into cRNA and labeled with Cy3-dCTP. Labeled cRNA was purified with RNeasy Mini columns (Qiagen). The quality of each cRNA sample was verified by total yield and specificity calculated with NanoDrop ND-1000 spectrophotometer measurements. 1.65 μg of labeled cRNA was used in each reaction and hybridization was carried out at 65°C for 17h in a hybridization oven rotator (Agilent). The arrays were washed using Agilent Gene expression washing buffers and Stabilization and Drying Solution, as suggested by the supplier. Slides were scanned on an Agilent microarray scanner (model G2565CA) and Agilent Feature Extraction software version 10.5.1.1 was used for image analysis. Gene expression data are available in the GEO database with the accession number: GSE52764.

Analysis of gene expression data

The Feature Extraction Software, which provided spot quality measures, was used to evaluate the quality and reliability of the hybridization. In particular, the flag "glsFound" (set to 1 if the spot had an intensity value significantly different from the local background and to 0 when otherwise) was used to filter out unreliable probes: the flag equal to 0 was to be noted as "null". Probes with a high proportion of "null" values were removed from the dataset in order to carry out a more solid, unbiased, statistical analysis. About forty percent (38%) of "null" was used as threshold in the filtering process, and a total of 22,523 available *Drosophila* transcripts were obtained.

Validation of relative gene expression by quantitative RT-PCR

Quantitative RT-PCR was used to validate the expression values of seven differentially expressed genes obtained from microarray experiments (Figure 2). 1µg of total RNA isolated from 30 heads flies (males) for *dSur KD* (*elav-Gal4/RNAi-104241*) and parental control strain (*RNAi-104241*) in two time points: "Pooled" (pooled samples every three hours over a 24h period) and "Night" (three hours in the dark phase, ZT15) was used to perform independent cDNA syntheses in a final volume of 10µl, using random hexamers and SuperScript II reverse transcriptase (Life Technologies). Three biological replicates were analysed. One µl aliquot of 1:100 diluted first-strand cDNA was PCR amplified in 10µl volume using the SYBR Green chemistry according to the manufacturer's recommendations (GoTaq qPCR Master Mix, Promega). Gene-specific primers (Supplementary Table 9) were designed using the Primer3 design tool to obtain 60-80 bp amplicons. A dissociation curve was used to confirm the specificity of the amplicon. We verified the efficiency of the primers by drawing standard curves for target genes and endogenous control (Rp49). PCR reactions were performed in triplicate in a 7500 Real-Time PCR System (Applied Biosystems). Thermal cycling conditions were as follows: 2 min denaturation at 95°C followed by 38 cycles for 25 sec denaturation at 95°C, 1 min annealing and elongation at 60°C, and a final 3 min elongation at 72°C. The $2^{-\Delta\Delta Ct}$ (RQ, relative quantification) method implemented in the 7500 Real Time PCR System software was used to calculate the relative expression ratio[4] 95% confidence intervals are associated to each time point.

ACKNOWLEDGMENTS

We thank all participants from all cohorts for donating their time and DNA. Svilen Stoynov and Magdalena Zaharieva for isolation of DNA from saliva samples and management of the online survey in Germany. We thank Dr. Giovana Dantas, Dr. Camila Morelatto, Dr Wolnei Caumo, Dr Iraci Torres for samples collection and cohort management in Brazil. We thank

Beniamina Pacchioni and Caterina Millino (MicroCribi Microarray Service, C.R.I.B.I., University of Padova, Italy) for their assistance with the microarray experiments, Marco Zanini for qRT-PCR experiments (Department of Biology, University of Padova, Italy) and Chiara Romualdi (Department of Biology, University of Padova, Italy) for support with bioinformatics programming and raw transcriptomic's data analysis.

SUPPLEMENTARY REFERENCES

1. Allebrandt KV, Amin N, Muller-Myhsok B, Esko T, Teder-Laving M, Azevedo RV *et al.* A K(ATP) channel gene effect on sleep duration: from genome-wide association studies to function in Drosophila. *Mol Psychiatry* 2013; **18**(1)**:** 122-132.

2. Levandovski R, Dantas G, Fernandes LC, Caumo W, Torres I, Roenneberg T *et al.* Depression scores associate with chronotype and social jetlag in a rural population. *Chronobiol Int* 2011; **28**(9)**:** 771-778.

3. Milani L, Leitsalu L, Metspalu A. An epidemiological perspective of personalized medicine: the Estonian experience. *J Intern Med* 2015; **277**(2)**:** 188-200.

4. Livak KJ, Schmittgen TD. Analysis of relative gene expression data using real-time quantitative PCR and the 2(-Delta Delta C(T)) Method. *Methods* 2001; **25**(4)**:** 402-408.
